# Systems Pharmacology Modeling Identifies a Novel Treatment Strategy for Bortezomib-Induced Neuropathic Pain

**DOI:** 10.3389/fphar.2021.817236

**Published:** 2022-01-19

**Authors:** Peter Bloomingdale, Cristina Meregalli, Kevin Pollard, Annalisa Canta, Alessia Chiorazzi, Giulia Fumagalli, Laura Monza, Eleonora Pozzi, Paola Alberti, Elisa Ballarini, Norberto Oggioni, Louise Carlson, Wensheng Liu, Mehrnoosh Ghandili, Tracey A. Ignatowski, Kelvin P. Lee, Michael J. Moore, Guido Cavaletti, Donald E. Mager

**Affiliations:** ^1^ Department of Pharmaceutical Sciences, School of Pharmacy and Pharmaceutical Sciences, University at Buffalo, The State University of New York, Buffalo, NY, United States; ^2^ Experimental Neurology Unit and Milan Center for Neuroscience, School of Medicine and Surgery, University of Milano-Bicocca, Monza, Italy; ^3^ Department of Biomedical Engineering, School of Science and Engineering, Tulane University, New Orleans, LA, United States; ^4^ Department of Immunology, Roswell Park Comprehensive Cancer Center, University at Buffalo, The State University of New York, Buffalo, NY, United States; ^5^ Department of Pathology and Anatomical Sciences, Jacobs School of Medicine and Biomedical Sciences, University at Buffalo, The State University of New York, Buffalo, NY, United States; ^6^ AxoSim, Inc., New Orleans, LA, United States; ^7^ Enhanced Pharmacodynamics, LLC, Buffalo, NY, United States

**Keywords:** bortezomib, dexanabinol, multiple myeloma, peripheral neuropathy, pharmacodynamics, systems pharmacology

## Abstract

Chemotherapy-induced peripheral neurotoxicity is a common dose-limiting side effect of several cancer chemotherapeutic agents, and no effective therapies exist. Here we constructed a systems pharmacology model of intracellular signaling in peripheral neurons to identify novel drug targets for preventing peripheral neuropathy associated with proteasome inhibitors. Model predictions suggested the combinatorial inhibition of TNFα, NMDA receptors, and reactive oxygen species should prevent proteasome inhibitor-induced neuronal apoptosis. Dexanabinol, an inhibitor of all three targets, partially restored bortezomib-induced reduction of proximal action potential amplitude and distal nerve conduction velocity *in vitro* and prevented bortezomib-induced mechanical allodynia and thermal hyperalgesia in rats, including a partial recovery of intraepidermal nerve fiber density. Dexanabinol failed to restore bortezomib-induced decreases in electrophysiological endpoints in rats, and it did not compromise bortezomib anti-cancer effects in U266 multiple myeloma cells and a murine xenograft model. Owing to its favorable safety profile in humans and preclinical efficacy, dexanabinol might represent a treatment option for bortezomib-induced neuropathic pain.

## Introduction

Chemotherapy-induced peripheral neuropathy (CIPN) is a common adverse side effect of cancer chemotherapy that occurs in approximately 30–40% of patients (Wolf et al., 2008; Staff et al., 2017). CIPN typically manifests as a tingling and numbness sensation in the extremities of the body, which can develop into a burning pain (Wolf et al., 2008). Muscle weakness, fatigue, and irreversible nerve damage can be life debilitating for many patients. Improvement in cancer therapy has resulted in a decrease in cancer death rates and an increase in the number of adult and pediatric cancer survivors who may struggle to manage long-term adverse side effects from treatment such as CIPN (Miaskowski et al., 2017; Kandula et al., 2018). An expert panel of the American Society of Clinical Oncology reported that there were no effective agents for preventing CIPN in cancer patients treated with neurotoxic chemotherapy based on a review of clinical trials conducted between 1946 to April 2013 (Hershman et al., 2014). Several nutraceuticals have been evaluated for their potential utility in CIPN, such as vitamin E, goshajinkigan, acetyl-L-carnitine, and alpha-lipoic acid; however, none were effective. Only one drug, duloxetine, has been moderately recommended to treat painful peripheral neuropathy (Hershman et al., 2014). Opioids, cannabis, and gabapentinoids have been used to treat neuropathic pain associated with CIPN. However, these symptomatic therapies exhibit limited efficacy, often associated with an addictive potential and adverse effects.

The molecular mechanisms of neurotoxicity by chemotherapeutics are multifactorial and complex, and there are a wide range of cellular components and processes that are disrupted. Some mechanisms may be exclusive to specific classes of chemotherapeutics (e.g., microtubule or proteasome inhibitors); however, many molecular pathways of toxicity are shared across neurotoxic agents. Some of the possible mechanisms involved in the pathogenesis of CIPN and neuropathic pain include mitochondrial changes, alterations in ion channels and ionic current, inflammation, oxidative and endoplasmic reticulum (ER) stress, activation of the intrinsic apoptosis pathway, MAPK pathway alterations, and the modulation of NMDA receptors (Jaggi and Singh, 2012). Mitochondrial dysfunction has emerged as a fundamental cause of CIPN, which consists of aberrant Ca^2+^ signaling, oxidative stress, and neuronal apoptosis. Mitochondria in the sensory axons of dorsal root ganglion (DRG) neurons become swollen and vacuolated when exposed to paclitaxel, oxaliplatin, and bortezomib. Opening of the mitochondrial permeability transition pore (mPTP) increases the permeability of the inner mitochondrial membrane resulting in a loss of membrane potential, mitochondrial swelling, decreased energy production, and rupture of the outer mitochondrial membrane, which initiates apoptosis through the release of pro-apoptotic proteins. Neuroinflammation is another important mechanism governing the development of CIPN (Lees et al., 2017). Upon peripheral nerve injury, resident macrophages, Schwann cells, satellite glial cells, and recruited immune cells secrete prostaglandins, chemokines, and cytokines. Neuroinflammatory mediators of the nerve microenvironment drive the complex interplay between cells of the neuroimmune system to promote adaptive (survival and growth) and maladaptive (neuropathic pain) responses. Several of the highly neurotoxic chemotherapeutics (paclitaxel, oxaliplatin, vincristine, and bortezomib) increase pro-inflammatory cytokines (TNFα and IL-1β) and downregulate anti-inflammatory cytokines (IL-10) in the DRG and spinal cord. Pro-inflammatory cytokines (TNFα, IL-1β, IL-6) and chemokines (CCL2) might be potential biomarkers and targets for predicting/preventing CIPN-related pain.

The multifactorial nature by which chemotherapeutics induce peripheral neurotoxicity warrants a systems pharmacology approach to evaluate the role of complex physiological processes involved in the development of CIPN and to identify potential treatments. In this study, a network-based systems pharmacology model of signal transduction and gene regulatory processes in peripheral neurons was constructed to predict the intracellular toxicodynamic effects of proteasome inhibitors and to identify drug targets for CIPN. Since the modulation of a single target will likely be inadequate for preventing CIPN, analyses that consider the entire topology and dynamics of the network were performed to identify combinatorial treatment strategies. A novel targeting approach was identified that consists of a TNFα inhibitor, NMDA receptor antagonist, and reactive oxygen species (ROS) inhibitor. Dexanabinol, a synthetic cannabinoid derivative, has been reported to inhibit all three targets (Eshhar et al., 1993; Nadler et al., 1993; Eshhar et al., 1995; Shohami et al., 1997). In this study, the neuroprotective effects of dexanabinol were evaluated in preclinical models of bortezomib-induced neurotoxicity.

## Materials and Methods

### Systems Pharmacology Model Development and Qualification

A bottom-up model development approach was utilized (Wang et al., 2012; Bloomingdale et al., 2018), and network construction was initiated using genes specific to drugs known to induce peripheral neuropathy, which were identified from a prior interaction network (Hur et al., 2014). A list of 230 genes was uploaded to DAVID and mapped to pathways in KEGG to identify significant gene-associated pathways. Signaling pathways were cross-referenced with literature to select pathways associated with peripheral neuropathy and not therapeutic indications. Three major pathways were selected (neurotrophin, mitogen-activated protein kinase, and apoptosis) as a foundational base network. Additional overlapping pathways were also included: TNFα, calcium signaling, PI3K-Akt, NFκB, p53, RAS, and protein processing in the endoplasmic reticulum. Other databases (e.g., Reactome and WikiPathways) were also used in model construction. Although peripheral neurons are post-mitotic, pathways related to the cell cycle and proliferation were included since aberrant cell cycle reentry can cause neuronal death.

Model qualification was achieved by comparing model predictions with an external transcriptomic dataset not used during network construction: Gene Expression Omnibus (GEO), GSE10470, in which gene expression was measured in neurons isolated from ATF4 knockout mice with and without exposure to homocysteate, an NMDA receptor agonist. Differentially expressed genes for each experimental condition were identified using Bioconductor packages (Biobase v2.30.0, GEOquery v2.40.0, limma v3.26.8). Data were log transformed using a base of two. Linear least squares regression was performed for each gene, and differentially expressed genes were identified using an empirical Bayes method with Benjamini and Hochberg FDR adjusted *p*-values. Network predictions were performed using normalized HillCube functions in *Odefy,* a MATLAB-based toolbox to convert Boolean models to a series of ordinary differential equations (Krumsiek et al., 2010). ATF4 and NMDA were fixed to zero and one to represent the gene knockout and presence of homocysteate. Simulations were performed until steady-state was reached. Network predictions for the change in node expression were reported as increasing when the initial state changes from 0 or 1 to a final state of 1, and decreasing when the initial state changes from 0 or 1 to a final state of 0. Network predictions were compared to the Log2 fold-change in differentially expressed genes. In cases where multiple probes exist for the same gene, the probe with the smallest adjusted *p*-value was selected for comparison. Averaging gene expression across multiple probes provided identical results.

### Network Model Analyses

Bortezomib was assumed to only inhibit the proteasome. Boolean logic functions were converted to normalized HillCube differential equations using *Odefy*, and default parameter values were used. Network simulations were performed for 25-time steps (arbitrary units), which was sufficient to reach steady-state.

Minimal intervention analysis (MIA) was performed using *CellNetAnalyzer*, a MATLAB-based toolbox. The intervention goal was to identify two- and three-node interventions that retain apoptosis in an OFF (zero) state in the presence of proteasome inhibition (proteasome fixed to zero). Nerve growth factor and brain derived neurotrophin factors were also assumed to be present (fixed to one).

An attractor analysis using a synchronous updating scheme and 10^6^ initial start states was performed for eight network perturbations. Proteasome was fixed to zero for all eight analyses to represent the effect of bortezomib. Relative activation frequencies were determined for all network components and apoptosis, which was calculated as the sum of node activation states in the attractors of each simulation divided by the total number of simulations performed. All analyses were performed using *BoolNet*.

### Neuronal and Multiple Myeloma Cell Culture

Neuronal SH-SY5Y cells were grown at 37°C and 5% CO_2_ in DMEM:F12 media supplemented with 10% fetal bovine serum (10,000 U/mL) and 1% penicillin-streptomycin (10,000 μg/ml) until a sufficient number of cells were obtained. SH-SY5Y cells were seeded at a density of 100,000 cells/well in 96 well plates and allowed to equilibrate for 24 h. Bortezomib (0.001, 0.01, 0.1, 1, 10, 100, 1,000 nM) and dexanabinol (1 and 10 μM) were added to their respective wells. Concentration ranges were selected in order to achieve a wide range around the IC_50_ of bortezomib and a potential therapeutic range of dexanabinol. Cell viability was measured using a colorimetric reagent (WST-1) (Berridge et al., 2005). After 48 h of drug exposure, 10% v/v of WST-1 was added to each well. Plates were briefly shaken and placed into an incubator for 2 h. Absorbance was measured using a SpectraMax 190 microplate reader at a wavelength of 450 nm (690 nm reference wavelength).

U266 myeloma cells were grown at 37°C and 5% CO_2_ in RPMI-1640 media supplemented with 15% fetal bovine serum (10,000 U/mL) and 1% penicillin-streptomycin (10,000 μg/mL) until a sufficient number of cells were obtained. U266 cells were seeded at a density of 25,000 cells/well in 96 well plates and were allowed to equilibrate for 24 h. Bortezomib (0.001, 0.01, 0.1, 1, 10, 100, 1,000 nM) and dexanabinol (1, 3, 10, 30, 100, 300 μM) were added to their respective wells, and concentration ranges were selected to achieve a wide range around the IC_50_ of each drug. Cell viability was measured after 24, 48, or 72 h of drug exposure as described above.

### Pharmacodynamic Modeling of Bortezomib and Dexanabinol *In Vitro* Cytotoxicity

For single agents, the decrease in cell viability (%) as a function of drug concentration was modeled using an inhibitory Hill function (Black and Leff, 1983). For the combinatorial effect of two drugs, cell viability was modeled using a modified version of the Ariens equation (Chakraborty and Jusko, 2002). An interaction parameter (Ψ_vit_) was incorporated on the bortezomib IC_50_ to account for synergistic (Ψ_vit_ < 1), antagonistic (Ψ_vit_ > 1), or additive (Ψ_vit_ = 1) effects on U266 cell viability. Parameters were estimated from single agent treatments and fixed while modeling interaction data to estimate Ψ_vit_.

### Dexanabinol Effects on LPS-Induced TNFα Production in Macrophages

WEHI-13VAR fibroblast cells, a TNFα sensitive cell line derived from a mouse fibrosarcoma, were grown in RPMI-1640 culture medium containing 2 mM L-glutamine, 10% fetal bovine serum, and 3 μg/ml gentamicin in T75 flasks at 37°C, 95% relative humidity, and 5% CO_2_. Cells were cultured to approximately 90% confluency and were always below passage 25 to avoid loss of TNFα sensitivity. Cells were prepared by detaching with 0.25% trypsin and 0.02% EDTA and resuspending in culture medium supplemented with 1 μg/ml actinomycin D to a concentration of 500,000 cells/ml. 100 µl of cell suspension were added to each well of a flat-bottom 96-well plate containing 100 µl of 2-fold serial dilutions of unknown samples, in duplicate, or known concentrations of rat recombinant TNFα standards in diluting medium (RPMI-1640, 2 mM L-glutamine, 1% fetal bovine serum, and 15 mM HEPES). Following 20 h of incubation, 10 µl of Cell Proliferation Reagent WST-1 (Roche Diagnostics, Indianapolis, IN, United States) in diluting medium was added to each well. WST-1 counting solution was used as a cell viability indicator, which was quantified spectrophotometrically (Berridge et al., 2005). After 4 h of incubation, absorbance at 440/700 nm was measured using a SpectraMax 96 microplate reader with SoftMax Pro v.4.0 acquisition and analysis software (MDS Analytical Technologies, Sunnyvale, CA, United States). A standard curve for absorbance (OD440–OD700) vs TNFα was plotted. The assay detection limit is approximately 1 pg/ml (Eskandari et al., 1990).

### Microphysiological Peripheral Nerve Model

All animal handling and tissue harvesting procedures were performed according to guidelines set by NIH and the Institutional Animal Care and Use Committee (IACUC) at Tulane University. DRG from an E15 rat litter were harvested, pooled, dissociated in trypsin, and passed through a 40 µM nylon mesh filter. Cells were then seeded in a 96 well ultra-low attachment treated spheroid microplate (Corning Inc., Corning, NY, United States) at a concentration of 45,000 cells per well in spheroid formation media composed of Neurobasal Medium supplemented with 2% v/v B27 supplement, 1% v/v GlutaMAX, 20 ng/ml nerve growth factor 2.5 S native mouse protein (NGF), and 1% v/v antibiotic/antimycotic solution (all from Thermo-Fisher Scientific, Waltham, MA, United States). Microplates were centrifuged at 500 g for 5 min and spheroids aggregate after overnight incubation at 37°C and 5% CO_2_.

DRG spheroids were seeded into dual-hydrogel constructs composed of a growth-restrictive outer-gel mold filled with a growth-permissive inner-gel culture scaffold. Outer-gel molds were fabricated as described previously, forming a long thin channel using a 10% w/v polyethylene glycol dimethacrylate (Polysciences Inc., Warrington, PA, United States), 1.1 mM lithium phenyl-2,4,6-trimethylbenzoylphosphinate (LAP; Allevi, Philadelphia, PA, United States), and 0.0001% w/v TEMPO (Millipore-Sigma, St. Louis, MO, United States) solution (Bowser and Moore, 2019). DRG spheroids were placed at one end of the channel. The outer gel mold was filled with a photo-translinkable, growth-permissive inner-gel solution composed of 4% w/v gelatin methacrylate (Allevi), 0.55 mM LAP, and 5.76% v/v 1-vinyl-2-pyrrolidone (Millipore-Sigma) supplemented with 0.004 mg/ml natural mouse laminin (Thermo-Fisher Scientific). Inner gels were cured with a 30 s exposure to 385 nm LED light (Bowser and Moore, 2019).

Completed dual-hydrogel DRG-spheroid cultures were incubated for 7 days in growth media composed of Basal Medium Eagle supplemented with 1% v/v Insulin-Transferrin-Selenium supplement (ITS), 1% v/v GlutaMAX, 2 mg/ml bovine serum albumin, 4 mg/ml glucose (Millipore-Sigma), 20 ng/ml NGF, and 1% antibiotic/antimycotic solution. Cultures were then matured 24–28 days in maturation media composed of growth media supplemented with 15% fetal bovine serum and 0.04 mg/ml ascorbic acid. All media components were purchased from Thermo-Fisher Scientific unless otherwise indicated. Fully-matured constructs were treated in maturation media for 48 h with one of the following six treatments: 1) 100 nM bortezomib (Millipore-Sigma) 2) 10 μM dexanabinol (Cayman Chemical, Ann Arbor, MI, United States) 3) 25 μM dexanabinol, 4) 10 μM dexanabinol + 100 nM bortezomib, 5) 25 μM dexanabinol + 100 nM bortezomib, or 6) vehicle alone (0.1% v/v DMSO; Millipore-Sigma).

Nerve impulse conduction was evaluated using extracellular field potential recording. All recordings were performed in continuously perfused, room temperature, bicarbonate-buffered, artificial cerebrospinal fluid (ACSF) bubbled with 95% O_2_, 5% CO_2_ as described previously (Huval et al., 2015). Electrical stimulation of nerve cultures and recording of compound action potential (CAP) propagation was performed similar to a previous description (Sharma et al., 2019). CAP propagation into the neuronal cell body cluster was recorded after nerite stimulation at two locations, 1.5 and 3 mm distal from the recording site. For each location, a stimulation pulse height of 15 V and pulse width of 200 μs was replicated ten times at 0.5 Hz and the ten resulting traces were averaged for analysis. Action potential amplitude (APA) is measured as the most negative potential recorded in the CAP trace. Recording distance was defined as the distance between the stimulating and recording electrodes. Latency to CAP onset was measured as the time elapsed between stimulation and the beginning of the CAP signal in the cell body cluster. Nerve conduction velocity (NCV) was defined as the recording distance divided by the latency to CAP onset.

### Bortezomib-Induced Peripheral Neuropathy Rat Model

A well-established bortezomib-induced peripheral neuropathy rat model was used to evaluate the neuroprotective effects of dexanabinol (Meregalli et al., 2010). Thirty younger adult female Wistar rats, (175–200 g; Envigo, Udine, Italy) randomized in 10 animals/group were used. Care and husbandry of animals were in conformity with the institutional guidelines in compliance with national (D.L. n. 26/2014) and international laws (EEC Council Directive 86/609, OJ L358, 1, Dec. 12, 1987; Guide for the Care and Use of Laboratory Animals, U.S. National Research Council, 1996). The experiments were examined and approved by the Ethics Committee of the University of Milano-Bicocca and Ministry of Health (approval number: 0007583/19 and 842/2018-PR, respectively). All rats were housed in a limited-access animal facility at the University of Milano-Bicocca, where room temperature, humidity, and artificial lighting were set to previously determined conditions. A veterinarian with specific expertise in animal studies and the authority to withdraw the animal from the experiment examined any animal showing signs of distress. Bortezomib and dexanabinol were purchased from LC Laboratories (Woburn, MA, United States, Canada) and Cayman Chemical Company (Ann Arbor, MI, United States).

Animals were allocated to experimental groups based on their baseline responses to the behavioral and neurophysiological studies, randomizing them into three groups containing 10 rats each: 1) dexanabinol vehicle-treated controls (vehicle) 2) bortezomib (Bort), and 3) dexanabinol + bortezomib (Dex + Bort). Dexanabinol was administered 30 min before bortezomib-injections at 9:00 am, and bortezomib or vehicle-treatments were performed between 9:30 to 11 am.

Dynamic and plantar aesthesiometry were performed under blinded conditions and were carried out by different researchers (C.M and L.M, respectively) between 9:00 to 12:00 am, as well as intraepidermal unmyelinated axons were counted in a blinded fashion (A.Ch). Fresh solutions were prepared before each administration. Bortezomib was dissolved in a solution of 10% Tween 80, 10% ethanol 100%, and 80% saline solution and was administered intravenously (IV) (0.2 mg/kg) three times a week for 8 weeks. Dexanabinol was formulated as a cremophor/ethanol solution (70%/30%) after a 1:20 dilution in saline. Dexanabinol was administered intraperitoneally (IP) (10 mg/kg) approximately 30 min prior to the administration of bortezomib. The general condition of the animals was assessed daily during the treatment period, and body weights were recorded twice weekly for toxicity assessment.

To assess the development of neurotoxicity sensory NCV and SAP amplitude were measured in caudal nerve using an electromyography apparatus (Myto2 ABN Neuro, Firenze, Italy) according to previous study (Monza et al., 2021). Briefly, distal caudal nerve was recorded orthodromically: recording cathode and anode were placed at 6 and 5 cm from the tip of the tail, the ground electrode at 2.5 cm from it, and stimulating anode and cathode respectively at 2 and 1 cm. Filters were set between 20 Hz and 3 KHz for sensory recordings. Animals were kept under deep isoflurane anesthesia, and body temperatures were kept constant at 34.5 ± 0.5°C for the whole procedure.

Pain sensitivity as a behavioral outcome was assessed prior to treatment (baseline) and after four and 8 weeks of BTZ-administration using the dynamic plantar aesthensiometer test (model 37450; Ugo Basile Biological Instruments, Comerio, Italy) and the dynamic plantar analgesiometer (model 37370; Ugo Basile Biological Instruments) (Chiorazzi et al., 2018). Mechanical allodynia was evaluated by quantifying the mechanical-escape behavior for 20 s after a metal filament was applied to the plantar skin of the hind paw according to methods as previously described (Chiorazzi et al., 2018). Mechanical threshold was automatically assessed by the mean value of six repeated application, measuring the paw withdrawal threshold (as withdrawal threshold expressed in grams) against stimulation with von Frey filaments, which exerted a progressively increasing puncture pressure reaching up to 50 g. Two hours after dynamic test evaluation, the response to noxious thermal stimulus was determined by a movable infrared radiant heat source (intensity of 40 IR), which was placed directly under the plantar surface on the hind paw and time taken for hind paw withdrawal was monitored (as withdrawal latency expressed in seconds). The nociceptive threshold response to thermal stimulus was determined by calculating the mean value of four repeated measurements. For both measures, a cut-off time of 25 s was used to prevent tissue damage in the absence of a response. All tests were operated by an experimenter blinded to groupings and in the controlled behavioral test room.

The damage of peripheral small nerve fibers, an analysis of intraepidermal nerve fiber (IENF) density, was performed at the end of the 8 weeks of drug treatment as previously described (Canta et al., 2016). Briefly, intraepidermal unmyelinated axons were counted in a blinded fashion, and the IENF density was obtained by immunochemistry analysis counting polyclonal anti-protein gene product 9.5 (PGP 9.5; GeneTex, Irvine, CA, United States)-positive fibers IENF/length of the epidermis (mm) (Canta et al., 2016).

### Multiple Myeloma Mouse Model

All animal protocols were approved by the Institutional Animal Care and Use Committee (IACUC) at Roswell Park Comprehensive Cancer Center. SCID mice were obtained from Laboratory Animal Resource of Roswell Park Comprehensive Cancer Center. Human multiple myeloma MM1.S cell line (gift from Dr Stephen Rosen, Chicago, IL, United States) was maintained in RPMI 1640 media containing 10% fetal bovine serum at 37°C under 5% CO_2_. Twenty SCID female mice at age of 4 weeks were irradiated with 300 Rad using a Mark II Cesium irradiator. Twenty-4 hours after irradiation, five million MM1.S cells were injected subcutaneously in the left ventral flank. Animals were monitored for tumor formation, and tumor sizes were measured with a digital caliper twice weekly. Tumor volumes were calculated using the modified ellipsoidal formula: tumor volume = 0.5 (length × width × width). When tumor volumes reached approximately 100 mm^3^, animals were divided to four groups with similar average tumor volumes. Treatments were administered twice a week via IP injection at the doses of 1 and 10 mg/kg for bortezomib and dexanabinol. The study was conducted for 4 weeks and mice were sacrificed when tumors grew to the limit enforced by IACUC.

### Pharmacokinetic and Pharmacodynamic Modeling

A PBPK model of bortezomib in mice (Zhang and Mager, 2015) was used to simulate a multiple dosing regimen of 1 mg/kg bortezomib administered IV to mice twice weekly. The average body weight of all mice in the bortezomib treatment group was used to determine the dose for each administration. Dexanabinol PK in mice has not been reported; therefore, dexanabinol PK in rats and humans (Brewster et al., 1997; Gershkovich et al., 2007) was allometrically scaled to predict mouse dexanabinol PK. A two-compartment PK model was fit to rat and human plasma concentrations of dexanabinol, and a power law relationship was used for parameter scaling:

$$X_{\mathrm{species}}=\alpha {\left(BW_{\mathrm{species}}\right)}^{\beta }$$ (1)

with *α* and *β* calculated for each PK model parameter (*X*) using species body weight (*BW*) (Boxenbaum, 1982). The values for *α* and *β*, along with the average body weight of mice in the dexanabinol treated group, were used to predict plasma dexanabinol PK in mice.

Bortezomib and dexanabinol PK were fixed when estimating pharmacodynamic model parameters. The rate of change of tumor volume (TV) was defined by the following differential equations:

$$\frac{dkg}{dt}=-kgr\times kg$$ (2)

$$\frac{dTV}{dt}=kg\left(t\right)\times TV-K_{\mathrm{Dex}}\times C_{\mathrm{Dex}}\times TV-K_{\mathrm{Bort}}\times e^{-\left(\psi_{viv}\times k_{\text{res}}\times t\right)}\times C_{Bort}\times TV$$ (3)

Tumor volume in the absence of drug was modeled using an exponential growth function (Norton and Simon, 1977). Drug effect was modeled as second-order elimination processes governed by the rate constants *K*_Bort_ and *K*_Dex_. A function that decreases the rate of elimination of tumor volume by bortezomib was included (*k*_res_), which improved model performance. The interaction term (*Ψ*_viv_) was incorporated on the parameter governing bortezomib resistance (*k*_res_). *Ψ*_viv_ was introduced to account for synergistic (*Ψ*_viv_ < 1), antagonistic (*Ψ*_viv_ > 1), or additive (*Ψ*_viv_ = 1) pharmacodynamic interactions. PK/PD modeling was performed using ADAPT 5 (D’Argenio et al., 2009).

### Statistical Analysis

Statistical analyses were performed using GraphPad Prism version 7.04. The change in nerve-on-a-chip NCV and APA for various concentrations of bortezomib was assessed using one-way ANOVA with Dunnet’s correction for multiple comparisons. Comparisons were made for each group with respect to the vehicle control. The change in nerve-on-a-chip NCV and APA for the drug combination was assessed using one-way ANOVA with Tukey’s correction for multiple comparisons. Drug pharmacodynamics on tumor volume in the multiple myeloma SCID mouse model relative to control was tested via multiple t-tests with Holm-Sidak Bonferroni correction (comparisons were made across all groups). IENF density, pain thresholds, and neurophysiological assessments in the rat model were analyzed using a Kruskal-Wallis test with Dunn’s correction for multiple comparisons across all groups.

## Results

### Systems Pharmacology Model Development and Qualification

A Boolean model of neuronal signaling was developed, which contains 131 nodes/components and 252 edges or reactions (Supplementary Figure S1). All model equations, initial conditions, and supporting references are provided in Supplementary Table S1. The model contains nine input nodes (BDNF, NGF, IL-1β, TFGβ, TNFα, IL-6, FasL, NMDA, and proteasome), which primarily consist of ligands that bind to 9 cell membrane receptors (NGFR, TrkA, TrkB, IL-1R, TFGβR, TNFαR, IL-6R, FasR, and NMDAR). Model inputs are connected to four endpoints (nerve damage, microtubule transport, apoptosis, and proliferation) through a complex intracellular network.

Network qualification was performed using a gene expression profile from an external microarray dataset of neurons isolated from ATF4 knockout mice with and without exposure to homocysteate, an NMDA receptor agonist. ATF4 is a central node in our network model and appears to be important component in oxidative/ER stress pathways. The ATF4 knockout provides a reasonable indirect dataset to test the connectivity of the final network model. ATF4 is downstream of NMDAR and is involved in the ROS signaling pathway, and the ATF4 dataset was considered suitable for model qualification purposes. Network-predicted changes in node activation states were compared to the Log_2_ fold-change in differentially expressed genes. There was a total of 2,113 differentially expressed genes between ATF4 knockout murine neurons exposed to an NMDA agonist and treatment naïve wild-type neurons. Nineteen genes that exhibited differential expression overlapped with nodes in the network model. Network predictions for the change in node expression were recorded as “increasing” when the initial state changed from 0 or 1 to a final state of 1 and “decreasing” when the initial state changed from 0 or 1 to a final state of 0. Qualitative model predictions agreed with the directionality of experimental observations for 16 out of 19 nodes (84%) (Supplementary Figure S2).

### Systems Model-Predicted Treatment Strategies for CIPN

Two types of network analyses, minimal intervention analysis (MIA) and attractor analysis, were performed to identify treatment strategies for proteasome-inhibitor induced peripheral neuropathy. MIA identified several targets/pathways, whereas attractor analysis enabled the prioritization of intervention targets based upon their relative importance.

MIA identified 224 potential combinatorial targets that might prevent neuronal apoptosis induced by proteasome inhibition. The potential strategies consisted of 109 two-target and 115 three-target interventions. The frequency of node interventions relative to the total number of intervention sets (up to two target combinations) are shown in Supplementary Figure S3A, and all two-target intervention sets are shown in Supplementary Figure S3B. Components of the TNFα signaling pathway, (i.e., TNFα, TNFαR, TRADD, and FADD) were the most frequently identified species. A TNFα inhibitor in combination with a pharmacological agent that modulates one of the 27 targets listed under *Target 2* in Supplementary Figure S3B is predicted to prevent proteasome inhibitor induced neuronal apoptosis. A TNFα inhibitor in combination with an inhibitor of ROS was identified as a two-target intervention set. The frequency of node interventions relative to the total number of intervention sets (up to three combinations) are shown in Supplementary Figure S3C. All three-target intervention sets are shown in Supplementary Figure S3D. Components of the TNFα signaling pathway were also prominent across the three-target intervention sets. At least one node in the TNFα signaling pathway (TNFα, TNFαR, TRADD, and FADD) was identified in 88 of the 115 three-target intervention sets. Three-target intervention sets are classified into five distinct groups (Supplementary Figure S3D), and the first group in particular consists of the inhibition of nodes in the TNFα signaling pathway, NMDA pathway (NMDA, NMDAR, NO, nNos), and MAPK pathway (MEKK1, MKK4, MKK7, TAK1).

**FIGURE 1:**
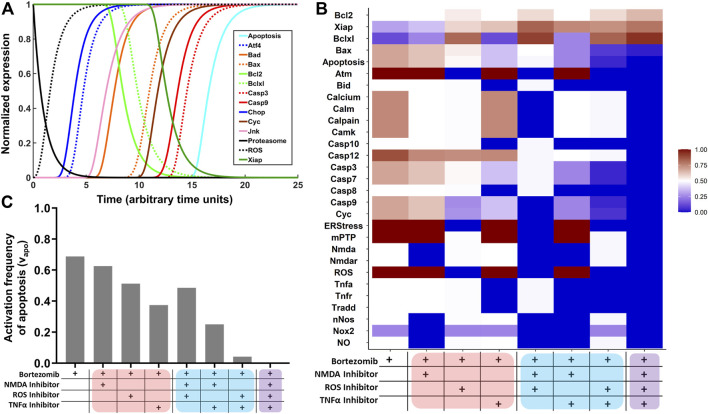
Network simulations to identify combinatorial treatment strategies for BIPN. **(A)** Network model simulations of select intracellular components in the presence of proteasome inhibition. The Boolean network model was converted to normalized HillCube differential equations using *Odefy*, and default parameter values were used (tau = 1; *k* = 0.5; *n* = 3). Simulations were performed for 25-time steps (arbitrary units). Results from attractor analysis for the relative activation frequency of **(B)** network nodes and **(C)** neuronal apoptosis across identified attractors for eight network perturbations: proteasome inhibition (bortezomib) in combination with a TNFα inhibitor, NMDA receptor antagonist, and/or ROS inhibitor. Monotherapies, two-target combinations, and the three-target combinations are highlighted in red, blue, and purple.

Network simulations were performed to assess the intraneuronal toxicodynamics of proteasome inhibition. Predicted normalized expression profiles of select cellular components is displayed in Figure 1A. Model predictions for all cellular components included in the network are shown in Supplementary Figure S4. Proteasome inhibition resulted in the production of ROS, which activated ER stress and the UPR. Several transcription factors increased in expression (e.g., ATF4 and CHOP), pro-survival proteins (Bcl-2, Bcl-xL, XIAP) decreased, and pro-apoptotic proteins (Bad, Bax, Cyc, Casp3, and Casp9) increased in expression. Lastly, proteasome inhibition resulted in the activation of neuronal apoptosis.

Based on TNFα and NMDA pathways being prominently featured in the MIA (Supplementary Figure S3), and simulations suggesting a role for ROS (Figure 1A), an attractor analysis was performed to predict the role of a TNFα inhibitor, NMDA receptor antagonist, and ROS inhibitor, alone and in combination, in decreasing the frequency of neuronal apoptosis (Figure 1B). For each treatment group, 10^6^ model simulations were performed, and the relative activation frequencies of network nodes are depicted as a heatmap. In the first column (bortezomib alone), nodes in the TNFα and NMDA pathways were equal to 0.5 and ROS was equal to 1. Hence, the TNFα/NMDA pathways are active at steady-state in half of the simulations, and the oxidative stress pathway is active at steady-state throughout all simulations. In columns 2–4, the relative activation frequencies of nodes in the NMDA, ROS, and TNFα pathways decrease to zero in the presence of their respective inhibitors. A trend of decreasing pro-apoptotic (Bax, Casp7, Casp3, Cyc, Casp9) and increasing pro-survival (Bcl-2, XIAP, Bcl-xL) species was observed. This trend becomes more pronounced for two-target combinations (columns 5–7) and is highly evident in the three-target combination (column 8). The relative activation frequency of neuronal apoptosis (ν_apoptosis_) was determined for the eight treatment arms (Figure 1C). ν_apoptosis_ was 0.69 in the bortezomib only group, which decreased to 0.63, 0.51, and 0.38 in the presence of single-agent treatments with an NMDA inhibitor, ROS inhibitor, or TNFα inhibitor. ν_apoptosis_ decreased further in the two-target combinations and was completely inhibited in the presence of all three inhibitors (Figure 1C).

### Assessment of Bortezomib and Dexanabinol *In Vitro* Cytotoxicity

**FIGURE 2:**
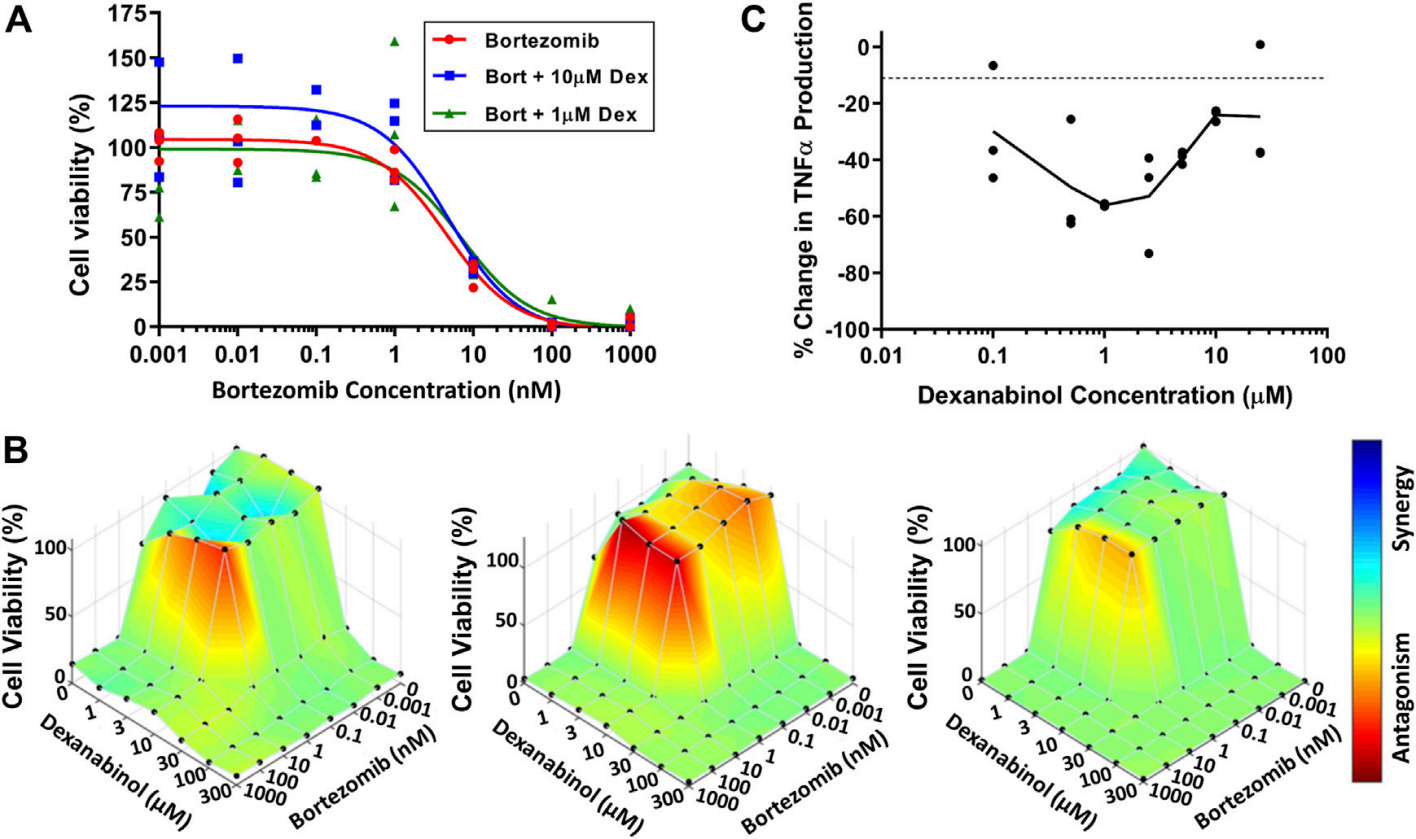
Assessment of *in vitro* cytotoxicity of bortezomib and dexanabinol. Cell viability was measured using WST-1. **(A)** Cytotoxicity of SH-SY5Y cells exposed to various concentrations of bortezomib in the absence (red) or presence of 1 μM (green) and 10 μM (blue) dexanabinol. **(B)** Cytotoxicity of U266 multiple myeloma cells exposed to a range of bortezomib and dexanabinol concentrations for (left) 24, (middle) 48, and (right) 72 h. Antagonistic, additive, and synergistic pharmacological relationships are colored in red, green, and blue. **(C)** Concentration-effect relationship of dexanabinol on LPS-induced TNFα production in primary rat macrophages.

SH-SY5Y neuroblastoma cells were used to assess the neuroprotective effect of dexanabinol on preventing bortezomib-induced cytotoxicity. The viability of SH-SY5Y cells at 48 h was determined for a range of bortezomib concentrations in the absence and presence of dexanabinol (1 and 10 μM; Figure 2A). A neuroprotective effect was not observed in SH-SY5Y cells, with the IC_50_ of bortezomib alone (4.40 nM) being comparable to the IC_50_ estimates in the presence of 1 and 10 μM of dexanabinol (7.00 and 4.62 nM). Greater concentrations of dexanabinol (≥25 μM) were shown to be cytotoxic (data not shown).

U266 multiple myeloma cells were used to assess the potential anti-cancer effects of dexanabinol and whether it would prevent bortezomib-induced cytotoxicity. The viability of U266 multiple myeloma cells was determined for a range of bortezomib and dexanabinol concentrations. A BLISS independence model was used to assess the nature of the pharmacological interaction between the two compounds at 24 (Figure 2B left), 48 (Figure 2B middle), and 72 (Figure 2B right) hours. The IC_50_ for dexanabinol ranged from 18.0 to 20.7 μM, and the IC_50_ for bortezomib ranged from 1.07 to 2.10 nM (Supplementary Tables S2–S4). An antagonistic relationship was observed around the IC_50_ of bortezomib for low concentrations of dexanabinol (≤10 μM) (Figure 2B). This antagonistic relationship appeared to be more pronounced at 24 and 48 h compared to 72 h. The time-dependent shift from an antagonistic to additive pharmacological relationship was quantified through a decrease in *Ψ*_vit_ from 2.71 (24 h) to 2.10 (48 h) to 1.40 (72 h) (Supplementary Tables S2–S4).

### Dexanabinol Inhibits Macrophage TNFα Production in a Biphasic Manner

WEHI-13VAR fibroblast cells were used to analyze supernatants from primary rat macrophage cultures for the presence of biologically active TNFα (Khabar et al., 1995; Ignatowski et al., 2000). Macrophages were stimulated with LPS to produce TNFα, which was inhibited in the presence of dexanabinol (Figure 2C). The concentration-effect relationship was bell-shaped, and maximal inhibition of TNFα occurred at 1 μM.

### Dexanabinol Exhibits Neuroprotective Effects at Low Concentrations Using Nerve-On-a-Chip

The efficacy of dexanabinol in preventing the neurotoxic effects of bortezomib was evaluated using a microphysiological model of a rat peripheral nerve, referred to as nerve-on-a-chip (Huval et al., 2015). Nerve conduction velocity (NCV) and action potential amplitude (APA) was measured for various concentrations of bortezomib to obtain a concentration-effect relationship (Supplementary Figure S5). Proximal and distal recordings of NCV and APA exhibited a decreasing trend for increasing concentrations of bortezomib. Proximal NCV at 1,000 nM of bortezomib decreased; however, this was not statistically significant (*p* = 0.054; Supplementary Figure S5C). There was a significant decrease in distal NCV after 48 h of bortezomib exposure at 100 and 1,000 nM (*p* < 0.05; Supplementary Figure S5D).

**FIGURE 3:**
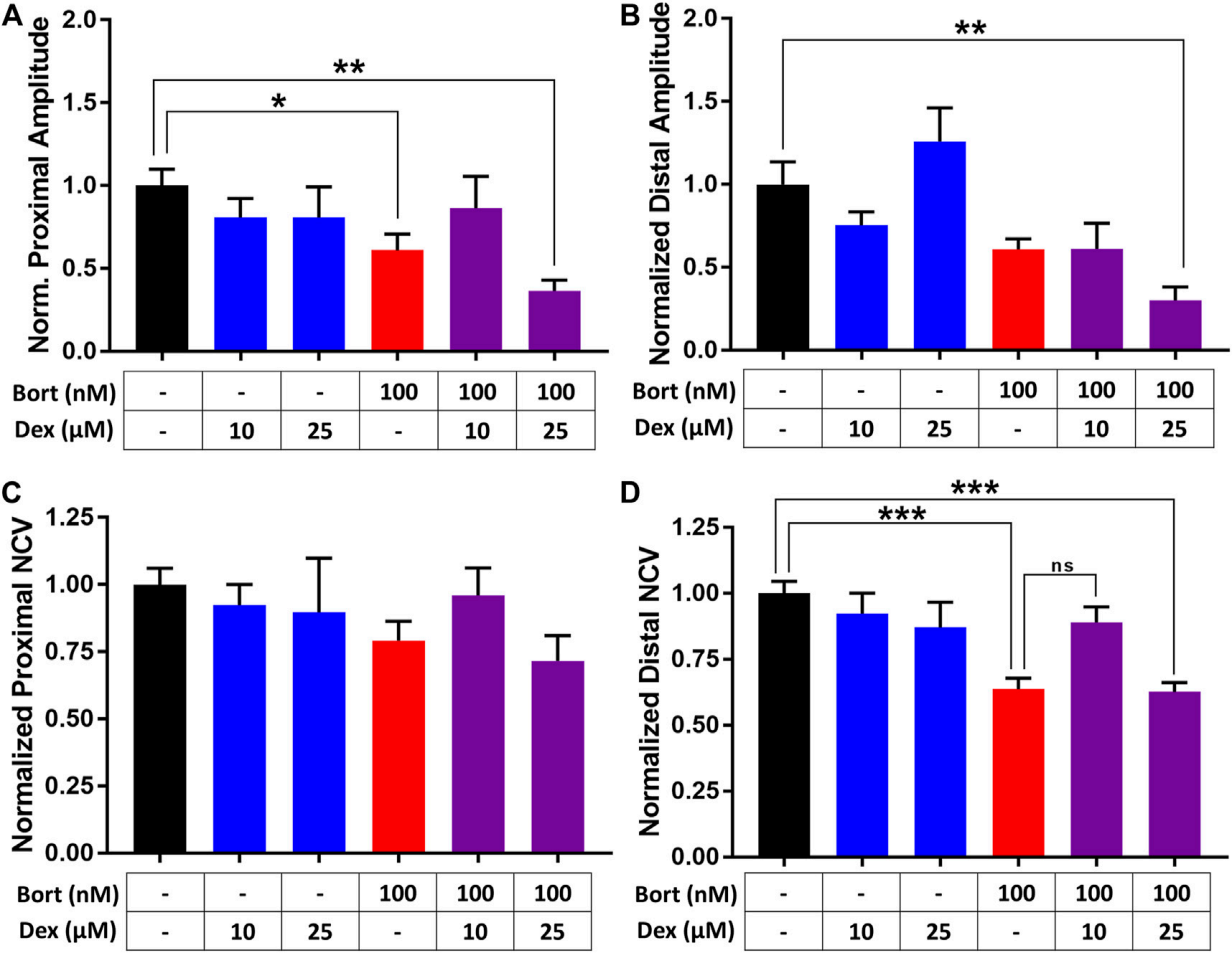
Assessment of dexanabinol neuroprotective effects using nerve-on-a-chip. The effect of dexanabinol on bortezomib-induced decreases in **(A)** proximal action potential amplitude (APA), **(B)** distal action potential amplitude, **(C)** proximal nerve conduction velocity, and **(D)** distal nerve conduction velocity. Action potential amplitudes and nerve conduction velocity were normalized to treatment naïve control (black). Dexanabinol (10 and 25 μM), bortezomib (100 nM), and the combination of both drugs are displayed as blue, red, and purple bars. GraphPad prism v7.04 was used to perform a one-way ANOVA with Tukey’s correction for multiple comparisons across all groups. Only significant comparisons to control are displayed. *p* values are reported as: ns (not significant), * (<0.05), ** (<0.01), and *** (<0.001).

The effects of dexanabinol on bortezomib-induced changes in APA (Figures 3A,B) and NCV (Figures 3C,D) were evaluated. Neurophysiological endpoints were normalized to their control values to account for batch-to-batch variability. The effects of 10 and 25 μM dexanabinol on NCV and APA were not significantly different from control, which indicates that dexanabinol is not neurotoxic at the selected concentrations. Nerves exposed to 100 nM of bortezomib for 48 h exhibited a significant decrease in the proximal amplitude and distal NCV (Figures 3A,D). The treatment group of 100 nM of bortezomib in combination with 10 μM of dexanabinol resulted in NCVs and amplitudes that were no longer significantly different than control. However, there was no significant difference observed in distal NCV between bortezomib alone and in combination with 10 μM of dexanabinol (*p* value = 0.070). The combination of bortezomib with a greater concentration of dexanabinol (25 μM) trended towards a further decrease in APA and NCV, which indicates a possible enhanced neurotoxic effect at greater dexanabinol concentrations. The biphasic inhibition of TNFα by dexanabinol (Figure 2C) might partially explain the improvement in proximal APA and distal NCV at lower dexanabinol exposure (10 μM) but not for the higher concentration (25 μM).

### Dexanabinol Prevents Bortezomib-Induced Allodynia and Thermal Hyperalgesia in Rats

**FIGURE 4:**
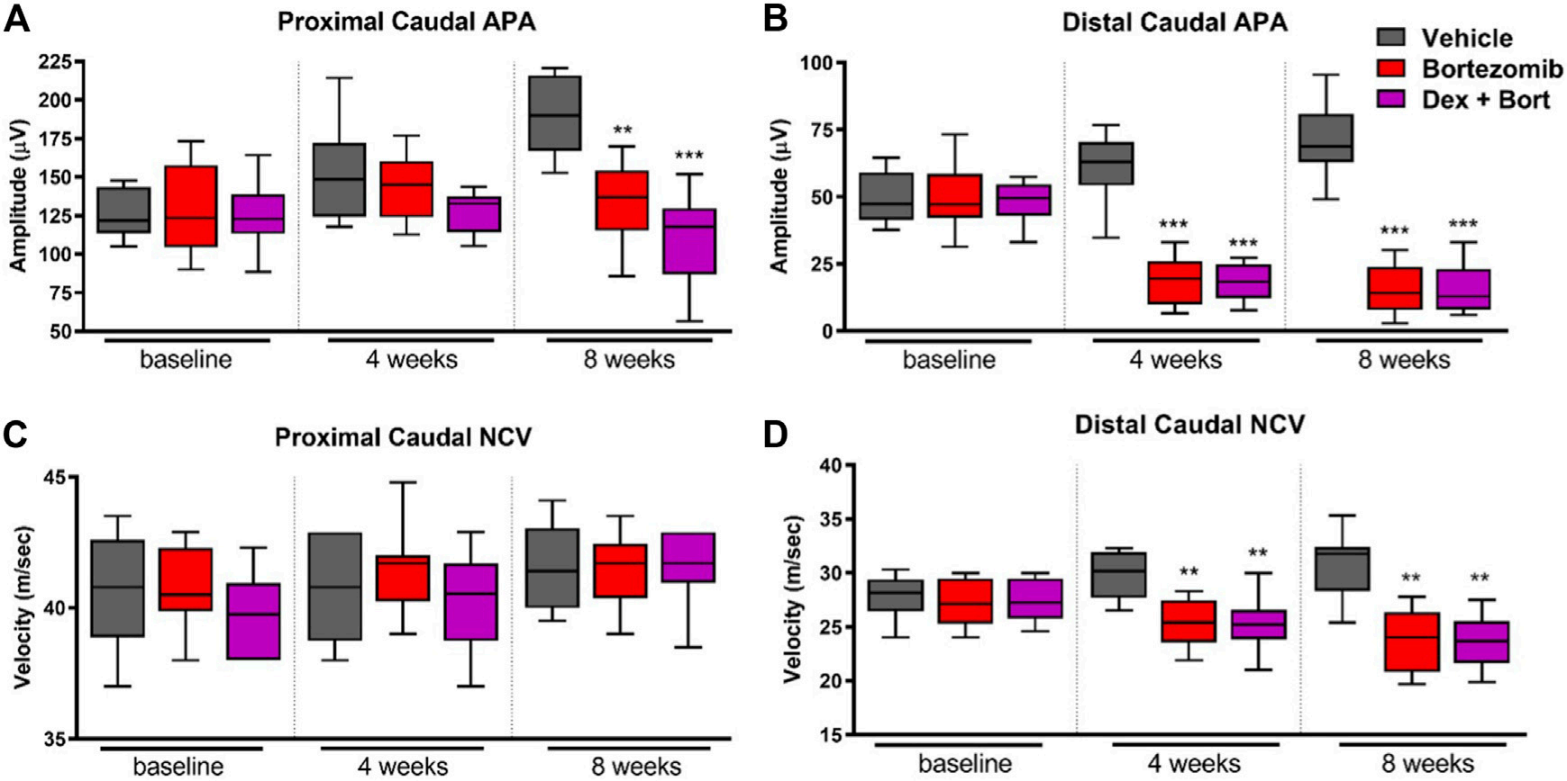
Nerve conduction studies of bortezomib and dexanabinol treatment in rats. Neurophysiological values were determined at baseline, 4 weeks of treatment, and 8 weeks of treatment. Top panels show neurophysiological changes in **(A)** proximal caudal SAP amplitude and **(B)** distal caudal SAP amplitude. Bottom panels show neurophysiological changes in **(C)** proximal caudal sensory NCV and **(D)** distal caudal sensory NCV. Bortezomib (Bort) was administered IV (0.2 mg/kg) three times a week, and dexanabinol (Dex) was administered IP (10 mg/kg) three times a week approximately 30 min prior to bortezomib. GraphPad prism v7.04 was used to perform a Kruskal-Wallis test with Dunn’s correction for multiple comparisons across all groups. Only significant comparisons to vehicle (Vehicle) are displayed. *p* values are reported as: * (<0.05), ** (<0.01), *** (<0.001).

The onset of peripheral neuropathy manifested after 4 weeks of chemotherapy with a significant decrease in distal, but not proximal, sensory action potential (SAP) amplitude (*p* < 0.001) and sensory NCV (*p* < 0.01) in bortezomib-treated groups compared to vehicle (Figure 4). Dexanabinol treatment did not prevent bortezomib-induced decreases in neurophysiological endpoints (Figure 4). After 8 weeks of bortezomib therapy, there were significant decreases in proximal caudal SAP amplitude (*p* < 0.01), distal caudal SAP amplitude (*p* < 0.001), and distal caudal sensory NCV (*p* < 0.01) (Figures 4A,B,D). The combination of dexanabinol and bortezomib resulted in further decreases in proximal caudal SAP amplitude than bortezomib alone with respect to vehicle (Figure 4A) (*p* < 0.001 vs *p* < 0.01). Similarly, co-treated groups with Dex + Bort showed a reduction of both distal caudal SAP (*p* < 0.001) and sensory NCV (*p* < 0.01) compared to vehicle (Figures 4B,D).

**FIGURE 5:**
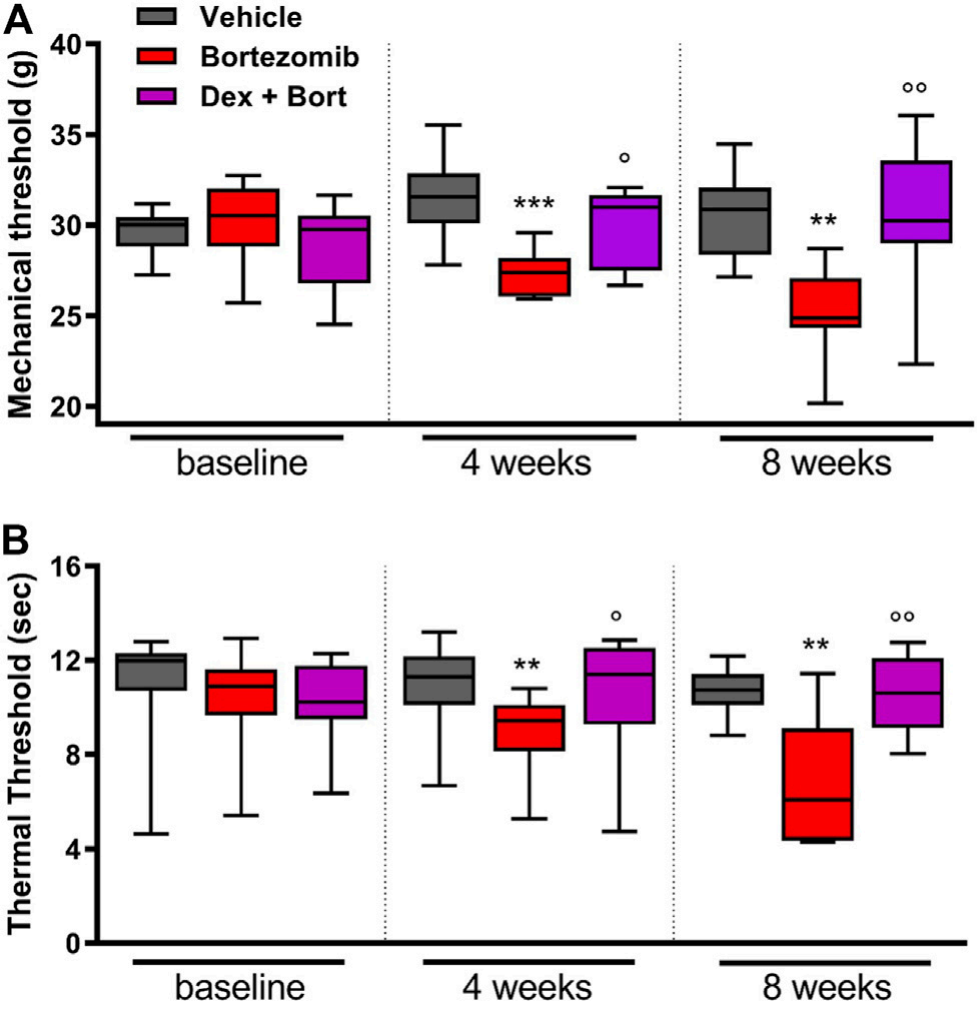
Efficacy of dexanabinol for preventing bortezomib-induced mechanical allodynia and thermal hyperalgesia in rats. Mechanical threshold (grams) was determined using dynamic test at baseline, 4 weeks of treatment, and 8 weeks of treatment **(A)**. The withdrawal latency to an infrared heat stimulus (seconds) was determined using a dynamic plantar analgesiometer at baseline, 4 weeks of treatment, and 8 weeks of treatment **(B)**. Bortezomib (Bort) was administered IV (0.2 mg/kg) three times a week, and dexanabinol (Dex) was administered IP (10 mg/kg) three times a week approximately 30 min prior to bortezomib. GraphPad prism v7.04 was used to perform a Kruskal-Wallis test with Dunn’s correction for multiple comparisons across all groups. *p* values are reported as: Bort vs vehicle (Vehicle), ** (<0.01), *** (<0.001) and Bort vs Dex + Bort, ○ (<0.05), ○ ○ (<0.01).

After four bortezomib-treatments, rats developed mechanical allodynia and thermal hyperalgesia, with a significant decrease in mechanical (*p* < 0.001; Figure 5A) and thermal (*p* < 0.01; Figure 5B) thresholds compared to vehicle. In addition, significant differences between bortezomib and the combination of bortezomib and dexanabinol were observed for mechanical (*p* < 0.05; Figure 5A) and thermal (*p* < 0.05; Figure 5B) thresholds. After 8 weeks of bortezomib therapy, significant decreases in mechanical (*p* < 0.01; Figure 5A) and thermal (*p* < 0.01; Figure 5B) thresholds compared to vehicle were observed. Dexanabinol therapy prevented bortezomib-induced mechanical allodynia and thermal hyperalgesia (Figures 5A,B). At 8 weeks, significant differences were observed between bortezomib and the combination of bortezomib and dexanabinol for mechanical (*p* < 0.01; Figure 5A) and thermal (*p* < 0.01; Figure 5B) thresholds.

**FIGURE 6:**
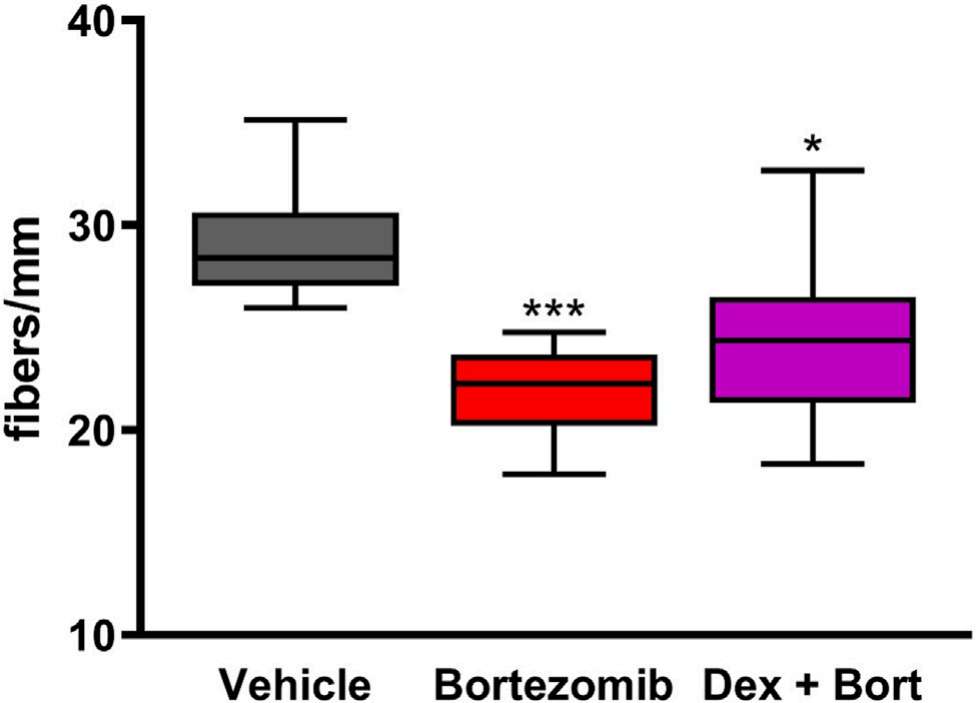
Efficacy of dexanabinol on bortezomib-induced decreases in intraepidermal nerve fiber (IENF) density. IENF density was measured at the end of the 8 weeks of drug treatment by an immunochemistry analysis using PGP 9.5. GraphPad prism v7.04 was used to perform a Kruskal-Wallis test with Dunn’s correction for multiple comparisons across all groups. Only significant comparisons to vehicle are displayed. *p* values are reported as: * (<0.05), *** (<0.001).

Bortezomib exposure for 8 weeks resulted in a significant decrease in intraepidermal nerve fibers (IENF) density (*p* < 0.001; Figure 6). Treatment with dexanabinol showed a trend toward improving the bortezomib-induced decrease of IENF density, although these changes were not statistically significant (Figure 6).

### Dexanabinol Does Not Interfere With the Anti-Cancer Activity of Bortezomib in a Multiple Myeloma Mouse Model

**FIGURE 7:**
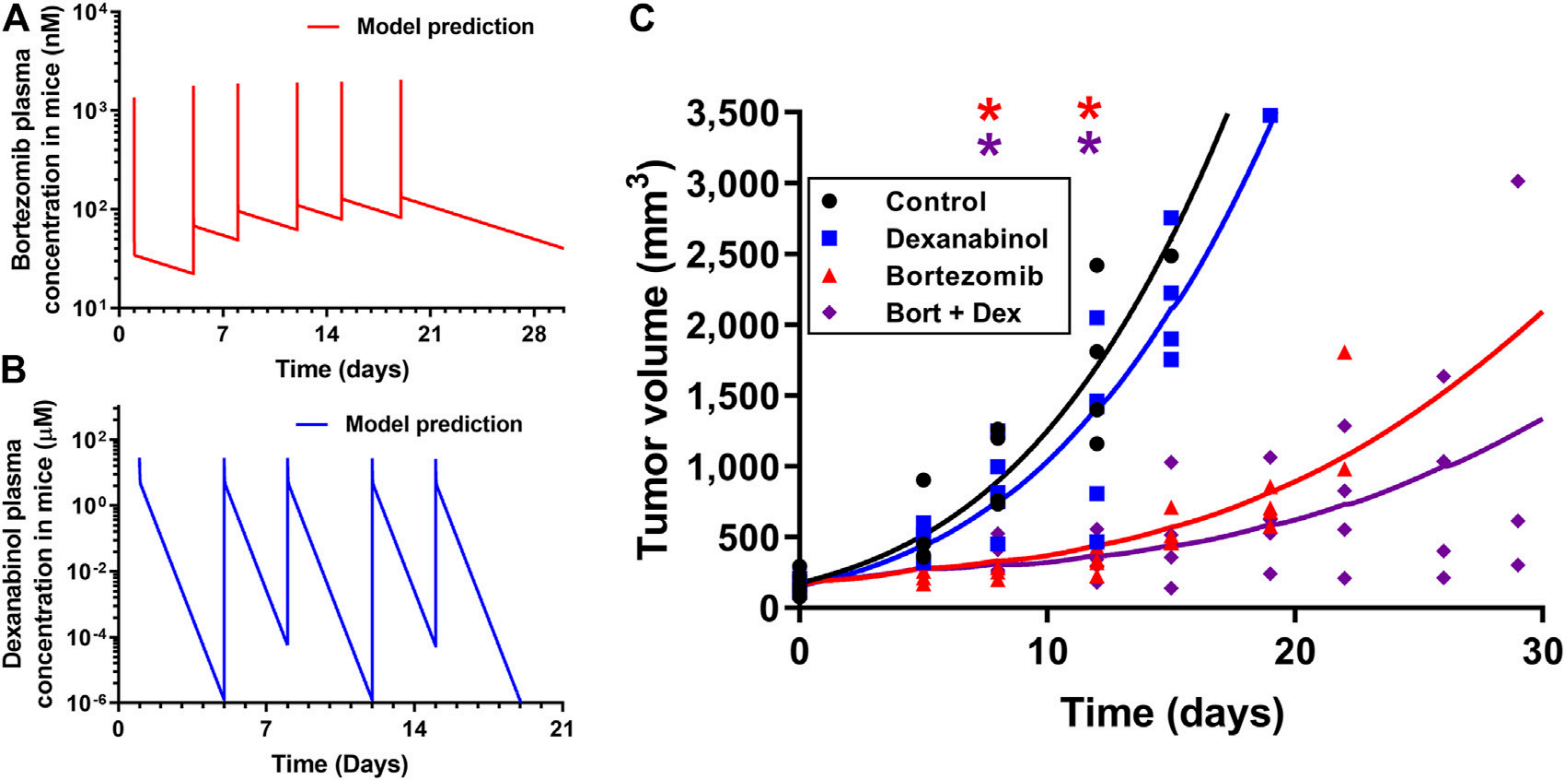
Pharmacokinetics and pharmacodynamics of dexanabinol and bortezomib in a MM1.S multiple myeloma mouse model. Model predicted mouse plasma concentrations of **(A)** 1 mg/kg of bortezomib administered on Days 1, 5, 8, 12, 15, and 19 and **(B)** 10 mg/kg of dexanabinol administered on Days 1, 5, 8, 12, and 15. **(C)** Pharmacodynamics of bortezomib and dexanabinol on MM1.S tumor volume in SCID mice. Tumors were grown to approximately 100 mm^3^ prior to drug administration. Mice (*n* = 20) were divided into four treatment groups: treatment naïve (black), dexanabinol (blue), bortezomib (red), and their combination (purple). Bortezomib and dexanabinol were administered IP twice weekly (1 and 10 mg/kg). Tumor volume measurements and pharmacodynamic model fitted profiles are displayed as solid markers and dashed lines. Asterisks indicate statistical significance from control (corrected *p* value < 0.5), which were obtained via multiple t-tests with Holm-Sidak Bonferroni correction (GraphPad Prism v7.04).

The pharmacodynamics of dexanabinol on the anti-cancer activity of bortezomib was investigated in a SCID MM1S multiple myeloma mouse model. Pharmacokinetic models were developed to predict the exposure of bortezomib and dexanabinol in mice, which were used to drive the pharmacodynamic effects on tumor volume. A previously developed physiologically-based pharmacokinetic (PBPK) model was used to predict mouse plasma concentrations of bortezomib (1 mg/kg) administered intravenously (IV) twice weekly (Figure 7A; Supplementary Table S5) (Zhang and Mager, 2015). The predicted PK profile exhibits a rapid distributive phase followed by a prolonged elimination phase. The predicted C_max_ after the last dose was 2.06 μM, and the trough concentration prior to the last dose was 0.083 μM. Dexanabinol PK in mice was scaled down using a model established using literature data from humans and rats. A two-compartment PK model was fit to plasma concentrations of dexanabinol in rats and humans (Supplementary Figure S6). Estimated PK parameters and the respective body weights of rats and humans were used to calculate allometric parameters (α and β), which were used to calculate PK parameters in mice (Supplementary Table S6). The predicted mouse plasma concentration-time profile of dexanabinol (10 mg/kg) administered intraperitoneally (IP) twice weekly is shown in Figure 7B. The predicted *C*_max_ after the last dose was 28.1 μM, and the model predicted half-lives of dexanabinol in humans, rats, and mice of 4.4, 4.1, and 10.8 h (Supplementary Table S6).

The pharmacodynamics of dexanabinol and bortezomib on tumor volume is shown in Figure 7C, and model parameter estimates are listed in Supplementary Table S7. Tumor volumes in the dexanabinol alone group were similar to control. Exposures to bortezomib alone and in combination with dexanabinol both resulted in statistically significant decreases in tumor volume. Dexanabinol did not antagonize the anti-cancer effects of bortezomib, and the combination trended towards improved tumor growth inhibition. The performance of the pharmacodynamic model improved after incorporating an exponential decay of the rate constant (*K*_bort_) governing the elimination of tumor volume by bortezomib exposure, suggesting acquired resistance. The exponential decay parameter (*k*_res_) was estimated to be 0.0076 h^−1^. An interaction term (*Ψ*_viv_) was placed on *k*_res_ and was estimated to be 0.883 with a 95% confidence interval of 0.80–0.97, which indicates a synergistic interaction by dexanabinol for slowing the decay rate of tumor volume elimination by bortezomib. Simulations using a prior signaling network model of multiple myeloma (Ramakrishnan and Mager, 2018) also qualitatively supports that dexanabinol does not impair the anti-cancer effect of bortezomib as tumor-associated apoptosis is predicted to remain active with the combination (Supplementary Figure S7).

## Discussion

CIPN remains an unmet medical need of increasing importance owing to the lack of available treatment options, the rising number of cancer survivors managing long-term adverse side effects of chemotherapy, and the need of non-opioid analgesics for the treatment of chronic pain (Manchikanti et al., 2012; Miaskowski et al., 2017; Staff et al., 2017). In this study, a systems pharmacology modeling approach enabled the identification of a potential treatment strategy for neuropathic pain associated with proteasome inhibitors, which was tested and confirmed in preclinical models of bortezomib-induced neurotoxicity.

A network-based systems pharmacology model (Supplementary Figure S1) provided a platform for exploring the molecular mechanisms involved in the pathogenesis of CIPN *in silico*, which identified potential treatment targets. TNFα was identified as an important signaling pathway that must remain inactive to prevent neuronal apoptosis. This supports the emerging concept of neuroinflammation as a major mechanism underlying the development of CIPN (Lees et al., 2017). Network analyses suggested that the combinatorial inhibition of TNFα, NMDAR, and ROS could prevent the activation of proteasome inhibitor-induced neuronal apoptosis. However, there could be potential clinical benefit for the modulation of single or dual targets. Anti-TNFα antibodies have shown to prevent allodynia in rats and partially restore sensory nerve APA in mice treated with bortezomib (Chiorazzi et al., 2013; Ale et al., 2014). In addition, bortezomib has been shown to induce neuropathic pain through the activation of presynaptic NMDA receptors in the spinal cord (Xie et al., 2017). Over the past decade there has been an increased interest in the use of ketamine, an NMDA receptor antagonist, for the treatment of neuropathic pain (Kamp et al., 2019). Several antioxidant therapies have also been investigated clinically for the treatment of CIPN; however, none of these agents have been effective as single agents (Wolf et al., 2008; Hershman et al., 2014). Duloxetine is another polypharmacological agent that has shown partial effectiveness in the treatment of CIPN (Thor and Katofiasc, 1995; Meng et al., 2019). The exact mechanisms of duloxetine neuroprotective effects are unclear, but the drug modulates alpha-1-adrenergic receptors, norepinephrine, serotonin, p38, and NF-kB. Although our final network model does not include all known or potential duloxetine mechanisms of action, the inhibition of p38/NF-kB were identified as potential targets (Supplementary Figure S3), which provides supporting evidence that the neuroprotective properties of duloxetine may be acting through this pathway (Meng et al., 2019). The combination of multiple therapeutics, such as duloxetine, anti-TNFα antibodies, ketamine, and antioxidants could be evaluated to treat CIPN. Alternatively, the generation of optimized polypharmacological drugs utilizing machine learning and mechanism-based modeling could enable the development of novel drugs for CIPN.

The lack of a clinically meaningful therapy for CIPN might result from the focus on single-target interventions rather than accounting for multiple etiological mechanisms. A cocktail of three single-target therapeutics would not likely be clinically viable; therefore, a single drug candidate with a polypharmacological profile was sought that matched the model-predicted combination strategy. Dexanabinol, a cannabinoid derivative, was selected for the treatment of BIPN based on its ability to inhibit TNFα, NMDAR, and ROS (Eshhar et al., 1995; Shohami et al., 1997). The drug also binds and affects several other (off) targets, and the *in vitro* pharmacodynamic potencies (EC_50_ and IC_50_ values) of dexanabinol for these targets are summarized in Supplementary Table S8 (Bloomingdale, 2018). Typical plasma dexanabinol concentrations in preclinical species and humans are greater than the reported *in vitro* potencies (Figure 7 and Supplementary Figure S6). Interest in targeting the endocannabinoid system for the treatment of CIPN has increased owing to preclinical and clinical efficacy in other sensory neuropathies (Abrams et al., 2007; Selvarajah et al., 2010; Blanton et al., 2019). Dexanabinol does not exhibit psychoactive effects and is not prone to cannabinoid receptor desensitization since it does not bind to cannabinoid receptors (Shohami et al., 1997). Dexanabinol was previously investigated for traumatic brain injury, but failed in phase III clinical development for a lack of efficacy (Maas et al., 2006). This could be due, in part, to the sensitive time window between the acute insult to the brain and drug administration. Nevertheless, dexanabinol exhibits a favorable pharmacokinetic profile and has been shown to be safe in humans (Brewster et al., 1997; Knoller et al., 2002; Maas et al., 2006).

In an effort to improve translatability over classical immortal cell lines, a combination of novel computational and experimental models was used to evaluate the potential neuroprotective effects of dexanabinol. The network-based systems pharmacology model provided an *in silico* projection of the neurotoxic effects of bortezomib and potential neuroprotective effects of dexanabinol on intraneuronal signaling processes. A 3D microphysiological model of peripheral nerves, although rat-derived, provides a better representation of *in vivo* physiology. Resident immune cells may be present in the peripheral nerve model; however, it lacks the chemotaxis and infiltration of peripheral immune cells and neuroimmune cells in the central nervous system, which have been increasingly recognized as important drivers of CIPN (Brandolini et al., 2019). Bortezomib has been shown to cause a dose-dependent decrease in neurite outgrowth in induced pluripotent stem cell (iPSC) derived neurons (Wing et al., 2017). We conducted a neurite outgrowth study using human iPSC-derived peripheral neurons (Peri.4U, Axiogenesis, Cologne, Germany); however, dexanabinol did not appear to prevent bortezomib-induced decreases in neurite outgrowth. Dexanabinol showed mixed, but promising effects in altering bortezomib-induced damage in the microphysiological nerve-on-a-chip, especially at lower concentrations (Figure 3). In addition, it restored pain thresholds that were significantly decreased in a rat model of BIPN (Figure 5), along with a partial recovery of the loss of IENF density induced by bortezomib at the end of experiment (Figure 6). Neurophysiological studies investigate only the population of large myelinated fibers, whereas behavioral tests and IENF explore unmyelinated and small-diameter myelinated fibers (i.e., those involved in neuropathic pain). Therefore, our data indicate that dexanabinol can attenuate (although not completely prevent) the effect of bortezomib, justifying the claim that it is a possible option for bortezomib-induced neuropathic pain. These *in vitro* and preclinical results support the hypothesis that dexanabinol may attenuate BIPN associated pain, which was generated from a systems pharmacological analysis of peripheral neuron signal transduction.

Once the potential beneficial effect of dexanabinol was confirmed *in vivo*, it was critical to determine whether it interferes with the ability of bortezomib to treat myeloma. To address this concern, the interaction of dexanabinol and bortezomib was assessed in preclinical models of multiple myeloma. Dexanabinol was shown not to compromise the anti-cancer effects of bortezomib in these experimental models (Figures 2B, 7C). The *in vitro* antagonistic effects observed in U266 multiple myeloma cells were transient and outside clinically relevant drug concentrations. Dexanabinol alone has been evaluated for the treatment of solid tumors, brain cancer, hepatocellular carcinoma, and pancreatic cancer (NCT01489826, NCT01654497, NCT02423239). Interestingly, dexanabinol appeared to enhance the anti-cancer effects of bortezomib in mice bearing MM1.S multiple myeloma xenografts (Figure 7C). Other drugs, such as lenalidomide and dexamethasone, which exhibit anti-TNFα properties, have also been given successfully with bortezomib for the treatment of multiple myeloma.

Quantitative and systems pharmacology (QSP) has emerged as an important discipline for evaluating disease and drug effects on biological systems in a holistic manner, providing useful tools and guiding experiments throughout various stages of drug discovery and development (Sorger et al., 2011). In this study, we used a general mathematical representation of bortezomib neurotoxic effects to identify a rational polypharmacological treatment strategy for CIPN. Complex diseases with multi-factorial etiologies, such as autoimmune and neurodegenerative disease, have not been effectively managed through single-target interventions. Hence, a rational polypharmacological approach that moves beyond Paul Erhlich’s magic bullet concept, a paradigm that has driven modern medicine for over a century, could lead to therapies for these unmet medical needs (Strebhardt and Ullrich, 2008; Barabasi et al., 2011). However, several study limitations must be acknowledged. First, peripheral neuropathy is a complex disorder and recapitulating the major signaling and physiological conditions are challenging. Although key intraneuronal pathways were featured in the computational model, there are other system components that may be important. As additional knowledge of the system accumulates, the signaling model could be refined to better evaluate intraneuronal toxicodynamics. Second, data availability and the translation of preclinical experiments to human therapeutic options are limited. The data used to develop and validate the systems pharmacology model were extracted from the public domain, and the original experimental conditions are often indirectly related (e.g., use of the ATF4 dataset for validation purposes). Compounds showing promise in animal experimental models of peripheral neuropathy may also fail in clinical settings. The nerve-on-a-chip model in this study utilized rat cells, and further development of microphysiological systems using human primary or iPSC-derived neurons might provide additional insights into species differences in outcomes (Kramer et al., 2020). Lastly, further research is needed to test and verify the mechanisms by which dexanabinol elicits its effects on CIPN *in vivo* and whether these preclinical results will ultimately translate to human therapeutics.

The systems pharmacology model (Supplementary Figure S1) could be expanded to incorporate additional components at the subcellular level as well as additional cell types (e.g., central and peripheral immune cells, satellite glial cells, and Schwann cells). Additionally, the model could be modified to capture quantitative relationships between system components (Eduati et al., 2017) and enable other applications, such as the ability to screen molecules with activity towards proteins in the model and support lead candidate selection. Interestingly, several components of the molecular network are also involved in central neurodegenerative diseases of the proteinopathy class, such as Alzheimer’s and Parkinson’s disease. For example, oxidative stress and inflammation are two hallmark characteristics of neurodegenerative disease (Fischer and Maier, 2015), and memantine, an NMDA receptor antagonist, is approved for the treatment of moderate-to-severe Alzheimer’s disease. This study highlights the use of a systems pharmacology approach to reposition a drug candidate for the treatment of neuropathic pain based upon its polypharmacological profile. Owing to its favorable pharmacokinetic and safety profile in humans and preclinical efficacy, dexanabinol might represent a clinically meaningful treatment for bortezomib-induced neuropathic pain.

## Data Availability

The original contributions presented in the study are included in the article/Supplementary Material; further inquiries can be directed to the co-corresponding authors.

## References

[B1] Abrams D. I. Jay C. A. Shade S. B. Vizoso H. Reda H. Press S. (2007). Cannabis in Painful HIV-Associated Sensory Neuropathy: a Randomized Placebo-Controlled Trial. Neurology. 68, 515–521. 10.1212/01.wnl.0000253187.66183.9c 17296917

[B2] Alé A. Bruna J. Morell M. Van De Velde H. Monbaliu J. Navarro X. (2014). Treatment with Anti-TNF Alpha Protects Against the Neuropathy Induced by the Proteasome Inhibitor Bortezomib in a Mouse Model. Exp. Neurol. 253, 165–173. 10.1016/j.expneurol.2013.12.020 24406455

[B3] Barabási A. L. Gulbahce N. Loscalzo J. (2011). Network Medicine: a Network-Based Approach to Human Disease. Nat. Rev. Genet. 12, 56–68. 10.1038/nrg2918 21164525 PMC3140052

[B4] Berridge M. V. Herst P. M. Tan A. S. (2005). Tetrazolium Dyes as Tools in Cell Biology: New Insights Into Their Cellular Reduction. Biotechnol. Annu. Rev. 11, 127–152. 10.1016/S1387-2656(05)11004-7 16216776

[B5] Black J. W. Leff P. (1983). Operational Models of Pharmacological Agonism. Proc. R. Soc. Lond. B Biol. Sci. 220, 141–162. 10.1098/rspb.1983.0093 6141562

[B6] Blanton H. L. Brelsfoard J. Deturk N. Pruitt K. Narasimhan M. Morgan D. J. (2019). Cannabinoids: Current and Future Options to Treat Chronic and Chemotherapy-Induced Neuropathic Pain. Drugs. 79, 969–995. 10.1007/s40265-019-01132-x 31127530 PMC8310464

[B7] Bloomingdale P. Nguyen V. A. Niu J. Mager D. E. (2018). Boolean Network Modeling in Systems Pharmacology. J. Pharmacokinet. Pharmacodyn. 45, 159–180. 10.1007/s10928-017-9567-4 29307099 PMC6531050

[B8] Bloomingdale P. (2018). Machine Learning and Network-Based Systems Toxicology Modeling of Chemotherapy-Induced Peripheral Neuropathy. Dissertation. Buffalo (NY): State University of New York at Buffalo. Appendix II, 309–316.

[B9] Bowser D. A. Moore M. J. (2019). Biofabrication of Neural Microphysiological Systems Using Magnetic Spheroid Bioprinting. Biofabrication. 12, 015002. 10.1088/1758-5090/ab41b4 31487700

[B10] Boxenbaum H. (1982). Interspecies Scaling, Allometry, Physiological Time, and the Ground Plan of Pharmacokinetics. J. Pharmacokinet. Biopharm. 10, 201–227. 10.1007/BF01062336 7120049

[B11] Brandolini L. D'angelo M. Antonosante A. Allegretti M. Cimini A. (2019). Chemokine Signaling in Chemotherapy-Induced Neuropathic Pain. Int. J. Mol. Sci. 20, 2904. 10.3390/ijms20122904 PMC6627296 31197114

[B12] Brewster M. E. Pop E. Foltz R. L. Reuschel S. Griffith W. Amselem S. (1997). Clinical Pharmacokinetics of Escalating i.V. Doses of Dexanabinol (HU-211), a Neuroprotectant Agent, in Normal Volunteers. Int. J. Clin. Pharmacol. Ther. 35, 361–365. 9314087

[B13] Canta A. Chiorazzi A. Carozzi V. A. Meregalli C. Oggioni N. Bossi M. (2016). Age-Related Changes in the Function and Structure of the Peripheral Sensory Pathway in Mice. Neurobiol. Aging. 45, 136–148. 10.1016/j.neurobiolaging.2016.05.014 27459934

[B14] Chakraborty A. Jusko W. J. (2002). Pharmacodynamic Interaction of Recombinant Human Interleukin-10 and Prednisolone Using *In Vitro* Whole Blood Lymphocyte Proliferation. J. Pharm. Sci. 91, 1334–1342. 10.1002/jps.3000 11977109

[B15] Chiorazzi A. Canta A. Meregalli C. Carozzi V. Sala B. Oggioni N. (2013). Antibody Against Tumor Necrosis Factor-α Reduces Bortezomib-Induced Allodynia in a Rat Model. Anticancer Res. 33, 5453–5459. 24324081

[B16] Chiorazzi A. Wozniak K. M. Rais R. Wu Y. Gadiano A. J. Farah M. H. (2018). Ghrelin Agonist HM01 Attenuates Chemotherapy-Induced Neurotoxicity in Rodent Models. Eur. J. Pharmacol. 840, 89–103. 10.1016/j.ejphar.2018.09.029 30268665

[B17] D’argenio D. Schumitzky A. Wang X. (2009). ADAPT 5 User’s Guide: Pharmacokinetic/Pharmacodynamic Systems Analysis Software. Los Angeles: Biomedical Simulations Resource.

[B18] Eduati F. Doldàn-Martelli V. Klinger B. Cokelaer T. Sieber A. Kogera F. (2017). Drug Resistance Mechanisms in Colorectal Cancer Dissected with Cell Type-Specific Dynamic Logic Models. Cancer Res. 77, 3364–3375. 10.1158/0008-5472.CAN-17-0078 28381545 PMC6433282

[B19] Eshhar N. Striem S. Biegon A. (1993). HU-211, a Non-Psychotropic Cannabinoid, Rescues Cortical Neurones from Excitatory Amino Acid Toxicity in Culture. Neuroreport. 5, 237–240. 10.1097/00001756-199312000-00013 8298080

[B20] Eshhar N. Striem S. Kohen R. Tirosh O. Biegon A. (1995). Neuroprotective and Antioxidant Activities of HU-211, a Novel NMDA Receptor Antagonist. Eur. J. Pharmacol. 283, 19–29. 10.1016/0014-2999(95)00271-l 7498309

[B21] Eskandari M. K. Nguyen D. T. Kunkel S. L. Remick D. G. (1990). WEHI 164 Subclone 13 Assay for TNF: Sensitivity, Specificity, and Reliability. Immunol. Invest. 19, 69–79. 10.3109/08820139009042026 2110931

[B22] Fischer R. Maier O. (2015). Interrelation of Oxidative Stress and Inflammation in Neurodegenerative Disease: Role of TNF. Oxid Med. Cell Longev. 2015, 610813. 10.1155/2015/610813 25834699 PMC4365363

[B23] Gershkovich P. Qadri B. Yacovan A. Amselem S. Hoffman A. (2007). Different Impacts of Intestinal Lymphatic Transport on the Oral Bioavailability of Structurally Similar Synthetic Lipophilic Cannabinoids: Dexanabinol and PRS-211,220. Eur. J. Pharm. Sci. 31, 298–305. 10.1016/j.ejps.2007.04.006 17560096

[B24] Hershman D. L. Lacchetti C. Dworkin R. H. Lavoie Smith E. M. Bleeker J. Cavaletti G. (2014). Prevention and Management of Chemotherapy-Induced Peripheral Neuropathy in Survivors of Adult Cancers: American Society of Clinical Oncology Clinical Practice Guideline. J. Clin. Oncol. 32, 1941–1967. 10.1200/JCO.2013.54.0914 24733808

[B25] Hur J. Guo A. Y. Loh W. Y. Feldman E. L. Bai J. P. (2014). Integrated Systems Pharmacology Analysis of Clinical Drug-Induced Peripheral Neuropathy. CPT Pharmacometrics Syst. Pharmacol. 3, e114. 10.1038/psp.2014.11 24827872 PMC4051377

[B26] Huval R. M. Miller O. H. Curley J. L. Fan Y. Hall B. J. Moore M. J. (2015). Microengineered Peripheral Nerve-On-A-Chip for Preclinical Physiological Testing. Lab. Chip. 15, 2221–2232. 10.1039/c4lc01513d 25850799

[B27] Ignatowski T. A. Kunkel S. L. Spengler R. N. (2000). Interactions between the Alpha(2)-Adrenergic and the Prostaglandin Response in the Regulation of Macrophage-Derived Tumor Necrosis Factor. Clin. Immunol. 96, 44–51. 10.1006/clim.2000.4877 10873427

[B28] Jaggi A. S. Singh N. (2012). Mechanisms in Cancer-Chemotherapeutic Drugs-Induced Peripheral Neuropathy. Toxicology. 291, 1–9. 10.1016/j.tox.2011.10.019 22079234

[B29] Kamp J. Van Velzen M. Olofsen E. Boon M. Dahan A. Niesters M. (2019). Pharmacokinetic and Pharmacodynamic Considerations for NMDA-Receptor Antagonist Ketamine in the Treatment of Chronic Neuropathic Pain: an Update of the Most Recent Literature. Expert Opin. Drug Metab. Toxicol. 15, 1033–1041. 10.1080/17425255.2019.1689958 31693437

[B30] Kandula T. Farrar M. A. Cohn R. J. Mizrahi D. Carey K. Johnston K. (2018). Chemotherapy-Induced Peripheral Neuropathy in Long-Term Survivors of Childhood Cancer: Clinical, Neurophysiological, Functional, and Patient-Reported Outcomes. JAMA Neurol. 75, 980–988. 10.1001/jamaneurol.2018.0963 29799906 PMC6142928

[B31] Khabar K. S. Siddiqui S. Armstrong J. A. (1995). WEHI-13VAR: a Stable and Sensitive Variant of WEHI 164 Clone 13 Fibrosarcoma for Tumor Necrosis Factor Bioassay. Immunol. Lett. 46, 107–110. 10.1016/0165-2478(95)00026-2 7590904

[B32] Knoller N. Levi L. Shoshan I. Reichenthal E. Razon N. Rappaport Z. H. (2002). Dexanabinol (HU-211) in the Treatment of Severe Closed Head Injury: a Randomized, Placebo-Controlled, Phase II Clinical Trial. Crit. Care Med. 30, 548–554. 10.1097/00003246-200203000-00009 11990913

[B33] Kramer L. Nguyen H. T. Jacobs E. Mccoy L. Curley J. L. Sharma A. D. (2020). Modeling Chemotherapy-Induced Peripheral Neuropathy Using a Nerve-On-A-Chip Microphysiological System. ALTEX. 37, 350–364. 10.14573/altex.2001181 32388569

[B34] Krumsiek J. Pölsterl S. Wittmann D. M. Theis F. J. (2010). Odefy--from Discrete to Continuous Models. BMC bioinformatics. 11, 233–310. 10.1186/1471-2105-11-233 20459647 PMC2873544

[B35] Lees J. G. Makker P. G. Tonkin R. S. Abdulla M. Park S. B. Goldstein D. (2017). Immune-Mediated Processes Implicated in Chemotherapy-Induced Peripheral Neuropathy. Eur. J. Cancer. 73, 22–29. 10.1016/j.ejca.2016.12.006 28104535

[B36] Maas A. I. Murray G. Henney H. Iii Kassem N. Legrand V. Mangelus M. (2006). Efficacy and Safety of Dexanabinol in Severe Traumatic Brain Injury: Results of a Phase III Randomised, Placebo-Controlled, Clinical Trial. Lancet Neurol. 5, 38–45. 10.1016/S1474-4422(05)70253-2 16361021

[B37] Manchikanti L. Helm S. Fellows B. Janata J. W. Pampati V. Grider J. S. (2012). Opioid Epidemic in the United States. Pain physician. 1538, ES9–38. 10.36076/ppj.2012/15/es9 22786464

[B38] Meng J. Zhang Q. Yang C. Xiao L. Xue Z. Zhu J. (2019). Duloxetine, a Balanced Serotonin-Norepinephrine Reuptake Inhibitor, Improves Painful Chemotherapy-Induced Peripheral Neuropathy by Inhibiting Activation of P38 MAPK and NF-Κb. Front. Pharmacol. 10, 365. 10.3389/fphar.2019.00365 31024320 PMC6465602

[B39] Meregalli C. Canta A. Carozzi V. A. Chiorazzi A. Oggioni N. Gilardini A. (2010). Bortezomib-induced Painful Neuropathy in Rats: a Behavioral, Neurophysiological and Pathological Study in Rats. Eur. J. Pain. 14, 343–350. 10.1016/j.ejpain.2009.07.001 19695912

[B40] Monza L. Fumagalli G. Chiorazzi A. Alberti P. (2021). Translating Morphology From Bench Side to Bed Side *via* Neurophysiology: 8-min Protocol for Peripheral Neuropathy Research. J. Neurosci. Methods. 363, 109323. 10.1016/j.jneumeth.2021.109323 34391792

[B41] Miaskowski C. Mastick J. Paul S. M. Topp K. Smoot B. Abrams G. (2017). Chemotherapy-Induced Neuropathy in Cancer Survivors. J. Pain Symptom Manage. 54, 204–e2. 10.1016/j.jpainsymman.2016.12.342 28063866 PMC5496793

[B42] Nadler V. Mechoulam R. Sokolovsky M. (1993). The Non-psychotropic Cannabinoid (+)-(3s,4s)-7-Hydroxy-delta 6- Tetrahydrocannabinol 1,1-dimethylheptyl (HU-211) Attenuates N-Methyl-D-Aspartate Receptor-Mediated Neurotoxicity in Primary Cultures of Rat Forebrain. Neurosci. Lett. 162, 43–45. 10.1016/0304-3940(93)90555-y 8121633

[B43] Norton L. Simon R. (1977). Growth Curve of an Experimental Solid Tumor Following Radiotherapy. J. Natl. Cancer Inst. 58, 1735–1741. 10.1093/jnci/58.6.1735 194044

[B44] Ramakrishnan V. Mager D. E. (2018). Network-Based Analysis of Bortezomib Pharmacodynamic Heterogeneity in Multiple Myeloma Cells. J. Pharmacol. Exp. Ther. 365, 734–751. 10.1124/jpet.118.247924 29632237 PMC5959840

[B45] Selvarajah D. Gandhi R. Emery C. J. Tesfaye S. (2010). Randomized Placebo-Controlled Double-Blind Clinical Trial of Cannabis-Based Medicinal Product (Sativex) in Painful Diabetic Neuropathy: Depression Is a Major Confounding Factor. Diabetes care. 33, 128–130. 10.2337/dc09-1029 19808912 PMC2797957

[B46] Sharma A. D. Mccoy L. Jacobs E. Willey H. Behn J. Q. Nguyen H. (2019). Engineering a 3D Functional Human Peripheral Nerve *In Vitro* Using the Nerve-On-A-Chip Platform. Sci. Rep. 9, 8921. 10.1038/s41598-019-45407-5 31222141 PMC6586937

[B47] Shohami E. Gallily R. Mechoulam R. Bass R. Ben-Hur T. (1997). Cytokine Production in the Brain Following Closed Head Injury: Dexanabinol (HU-211) Is a Novel TNF-Alpha Inhibitor and an Effective Neuroprotectant. J. Neuroimmunol. 72, 169–177. 10.1016/s0165-5728(96)00181-6 9042110

[B48] Sorger P. K. Allerheiligen S. R. Abernethy D. R. Altman R. B. Brouwer K. L. Califano A. (2011). “Quantitative and Systems Pharmacology in the post-genomic Era: New Approaches to Discovering Drugs and Understanding Therapeutic Mechanisms,” in An NIH white paper by the QSP workshop group (Bethesda, MD: NIH White Paper by the QSP Workshop Group).

[B49] Staff N. P. Grisold A. Grisold W. Windebank A. J. (2017). Chemotherapy-Induced Peripheral Neuropathy: A Current Review. Ann. Neurol. 81, 772–781. 10.1002/ana.24951 28486769 PMC5656281

[B50] Strebhardt K. Ullrich A. (2008). Paul Ehrlich's Magic Bullet Concept: 100 Years of Progress. Nat. Rev. Cancer. 8, 473–480. 10.1038/nrc2394 18469827

[B51] Thor K. B. Katofiasc M. A. (1995). Effects of Duloxetine, a Combined Serotonin and Norepinephrine Reuptake Inhibitor, on Central Neural Control of Lower Urinary Tract Function in the Chloralose-Anesthetized Female Cat. J. Pharmacol. Exp. Ther. 274, 1014–1024. 7636716

[B52] Wang R. S. Saadatpour A. Albert R. (2012). Boolean Modeling in Systems Biology: an Overview of Methodology and Applications. Phys. Biol. 9, 055001. 10.1088/1478-3975/9/5/055001 23011283

[B53] Wing C. Komatsu M. Delaney S. M. Krause M. Wheeler H. E. Dolan M. E. (2017). Application of Stem Cell Derived Neuronal Cells to Evaluate Neurotoxic Chemotherapy. Stem Cell Res. 22, 79–88. 10.1016/j.scr.2017.06.006 28645005 PMC5737666

[B54] Wolf S. Barton D. Kottschade L. Grothey A. Loprinzi C. (2008). Chemotherapy-Induced Peripheral Neuropathy: Prevention and Treatment Strategies. Eur. J. Cancer. 44, 1507–1515. 10.1016/j.ejca.2008.04.018 18571399

[B55] Xie J. D. Chen S. R. Chen H. Pan H. L. (2017). Bortezomib Induces Neuropathic Pain Through Protein Kinase C-Mediated Activation of Presynaptic NMDA Receptors in the Spinal Cord. Neuropharmacology. 123, 477–487. 10.1016/j.neuropharm.2017.06.027 28663117 PMC5546238

[B56] Zhang L. Mager D. E. (2015). Physiologically-Based Pharmacokinetic Modeling of Target-Mediated Drug Disposition of Bortezomib in Mice. J. Pharmacokinet. Pharmacodyn. 42, 541–552. 10.1007/s10928-015-9445-x 26391023 PMC4620045

