# Supplementary material for: Systems Pharmacology Modeling Identifies a Novel Treatment Strategy for Bortezomib-Induced Neuropathic Pain
Source: Front Pharmacol. 2022 Jan 19;12:817236. doi: 10.3389/fphar.2021.817236 (PMC8809372; doi:10.3389/fphar.2021.817236)
Supplement: Supplementary file 1 [file DataSheet1.docx]

Supplementary Material

# Supplementary Figures and Tables

## Supplementary Figures


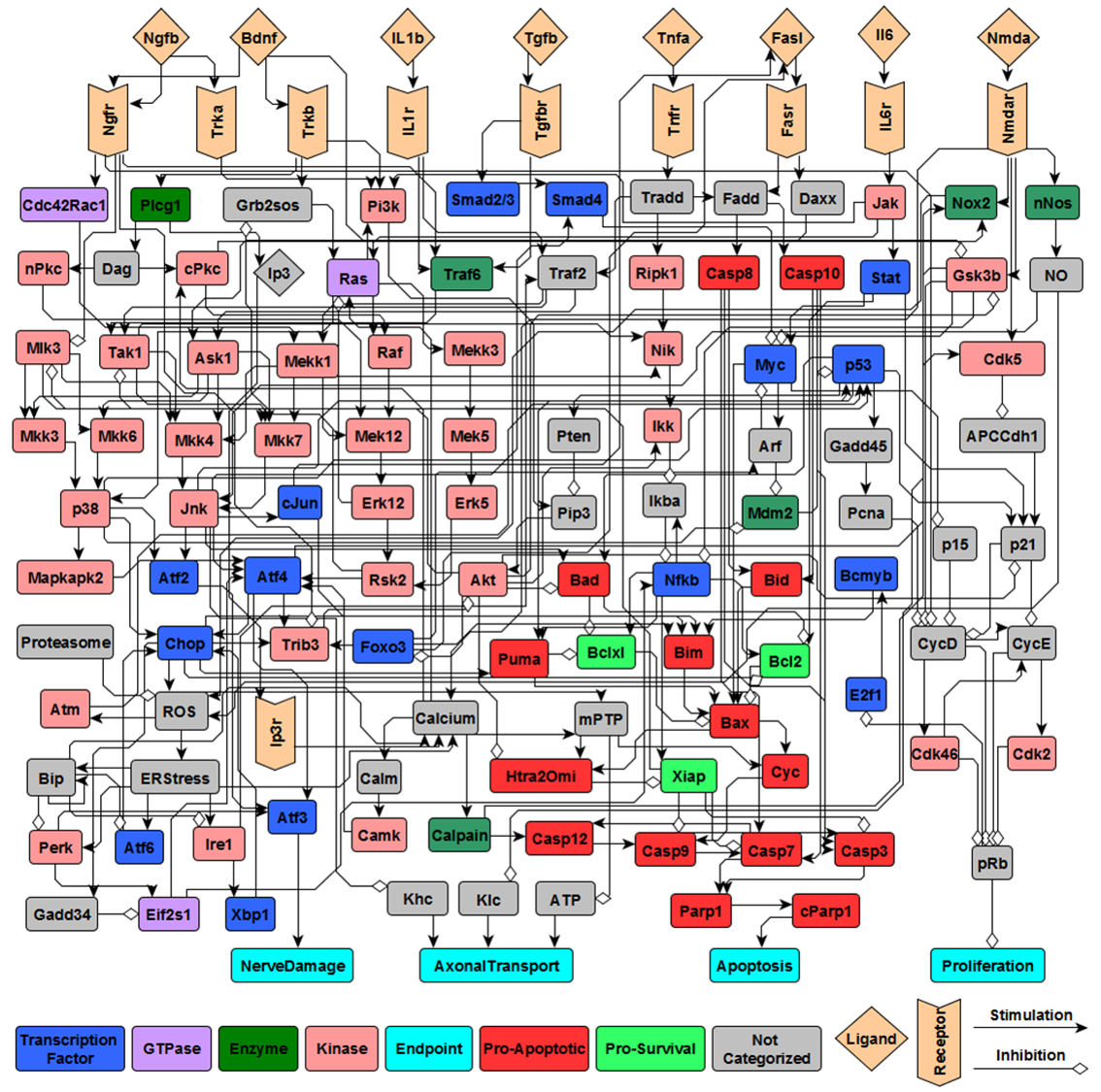


**Supplementary Figure S1.** **Network-based systems pharmacology model of intracellular signaling in peripheral neurons.** Nodes: transcription factors (blue), GTPases (purple), enzymes (dark green), kinases (pink), phosphatases (green), endpoints (light blue), pro-apoptosis (red), pro-survival (lime green), not categorized (gray), ligands (tan diamond), receptors (tan rotated flags). Edges: stimulatory edge (black arrowhead) and inhibitory edge (white diamond). Components of the neurotrophin signaling pathway are included (top left), and activation of nerve growth factor receptor (NGFR; p75NTR) and tropomyosin receptor kinases (TrkA/TrkB) by NGF and BDNF propagates signals through the network to activate downstream pathways critical for cell survival (Brunet et al., 2001;Nusser et al., 2002). Components of the MAPK pathway were organized in a hierarchical manner (middle left) based upon a three-tier signaling cascade. The NMDA pathway (top right) allows for NMDA agonists, such as glutamate, to bind NMDA receptors (NMDAR) and increase intracellular Ca^2+^ ­and ROS (MacDermott et al., 1986;Moriyoshi et al., 1991;Girouard et al., 2009;Choi et al., 2016). Inhibition of the proteasome leads to the accumulation of intracellular proteins and the production of oxidative stress (Ding and Keller, 2001), which induces ER stress and triggers the unfolded protein response (UPR). The network also contains three proinflammatory cytokines and receptors (IL-1β/ IL-1R, IL-6/ IL-6R, and TNFα/ TNFαR). Proinflammatory cytokines are input nodes, which physiologically represent a neuronal microenvironment created by surrounding peripheral neuroimmune system components (e.g., Schwann cells, damaged neurons, macrophages, and other leukocytes). The apoptosis pathway is also incorporated (bottom right), which can be activated through extrinsic (TNFα and FasL) and intrinsic (mPTP) pathways.


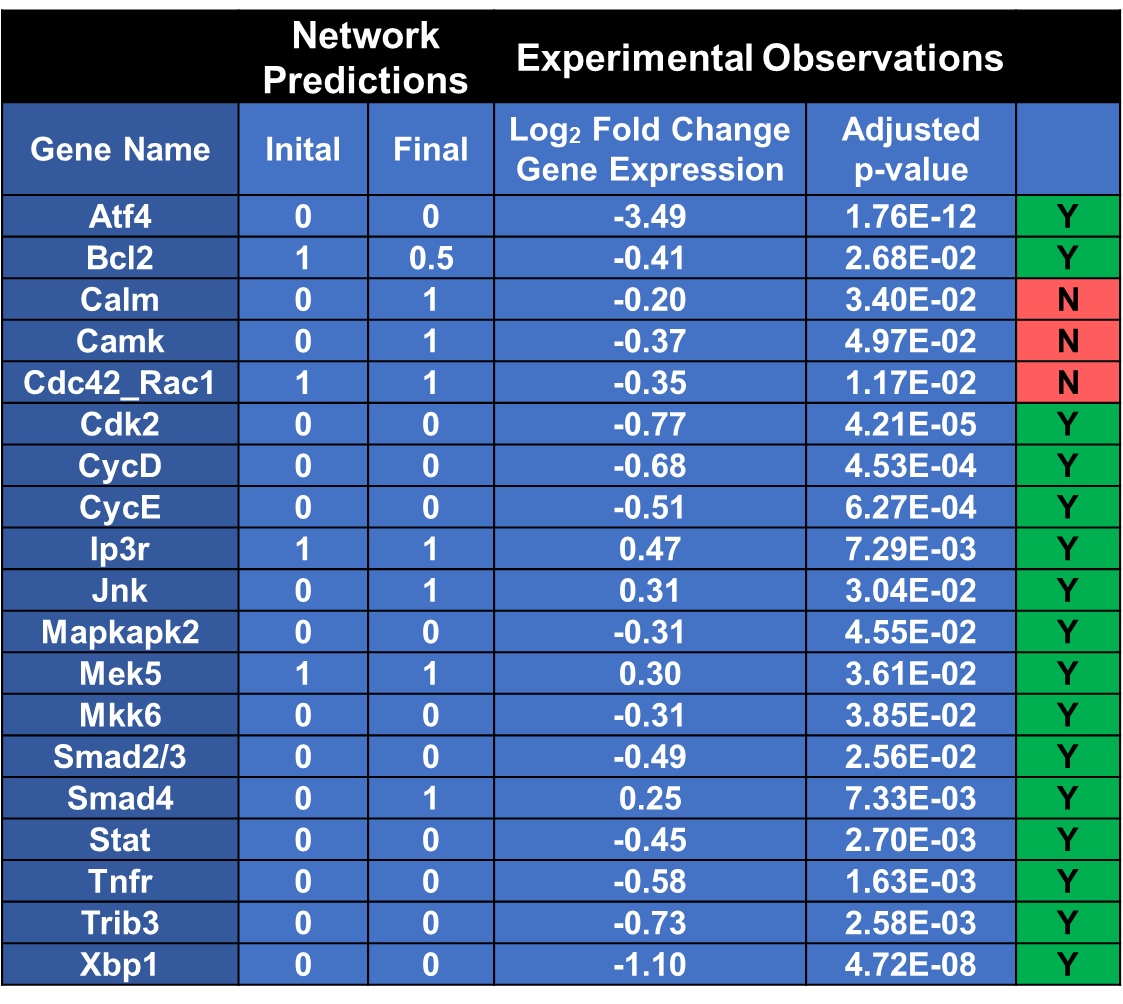


**Supplementary Figure S2.** **Boolean network predictions compared against an experimental microarray dataset of ATF4 knockout murine neurons exposed to an NMDA agonist (GSE10470).** Model predictions for 16 of 19 nodes agree with experimental observations. Y = yes (agree); N= no (disagree).


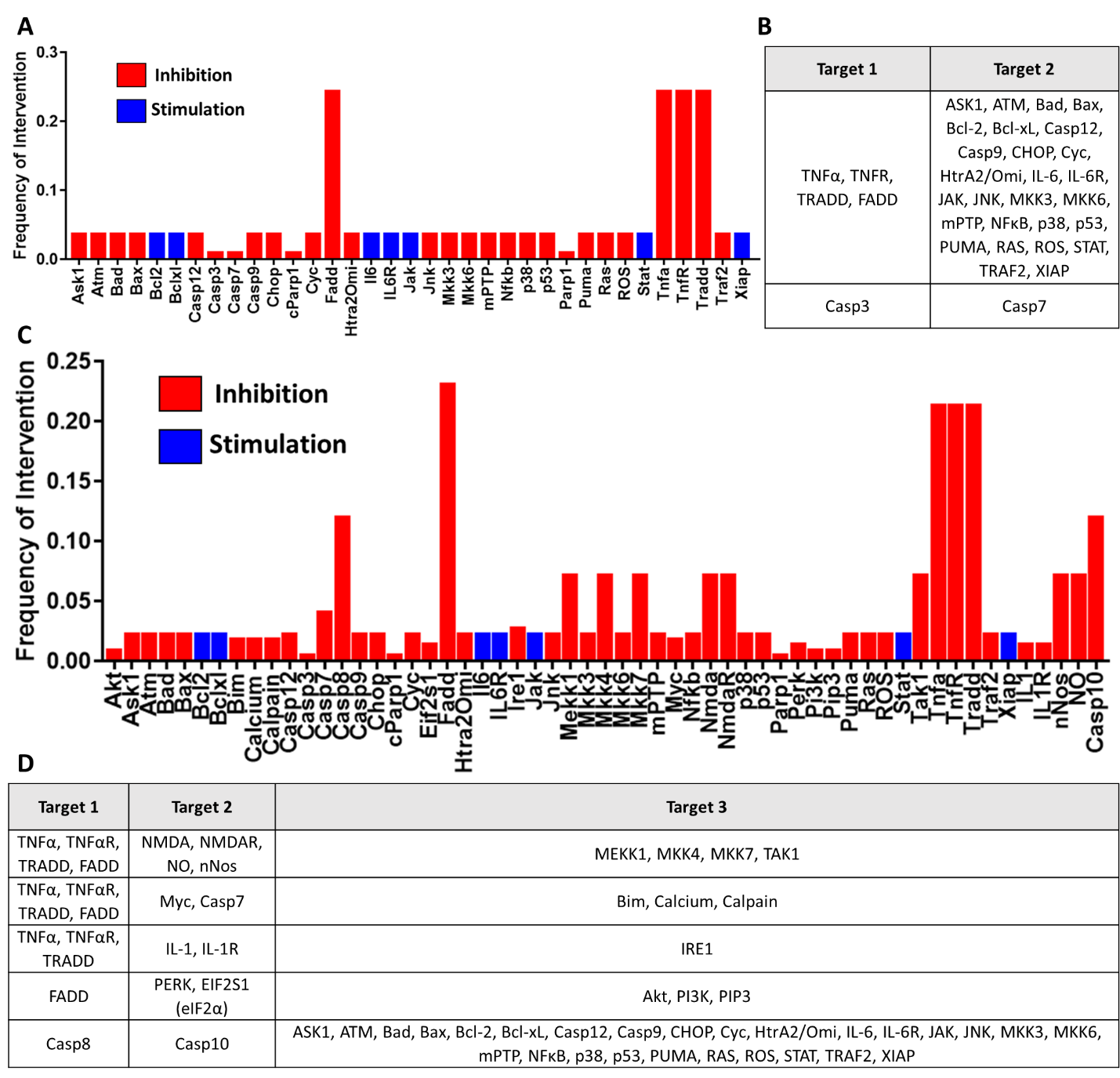


**Supplementary Figure S3.** **Combinatorial target strategies identified through minimal intervention analysis.** (A) Intervention frequency for up to two targets. (B) Combinatorial target strategies for 109 two-target interventions. (C) Intervention frequency for up to three targets. (D) Combinatorial treatment strategies for 115 three-target interventions. Intervention frequencies (y-axis) for network nodes (x-axis) are displayed relative to the total number of intervention sets. Red and blue bars indicate node inhibition and stimulation. Tables represent network-predicted combinations of two- or three-target interventions that prevent neuronal apoptosis in the presence of proteasome inhibition.


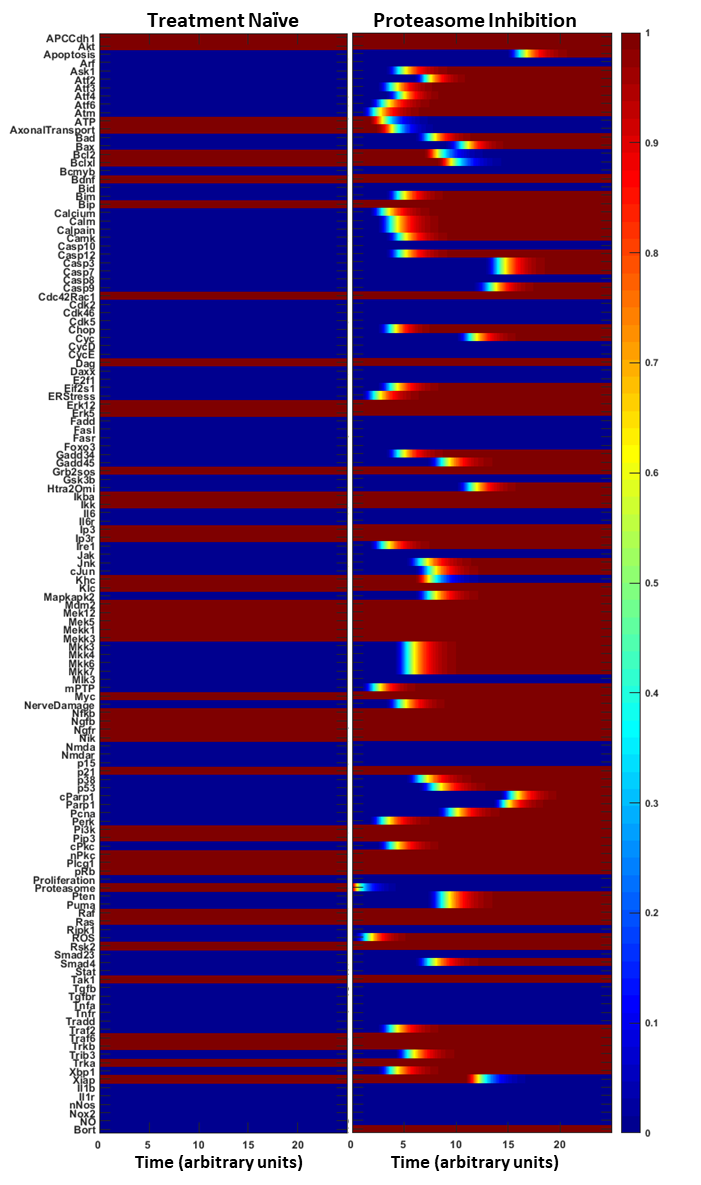


**Supplementary Figure S4.** **Network model simulations of intraneuronal signaling in the absence (left column) and presence (right column) of proteasome inhibition.** The Boolean network model was converted to normalized HillCube differential equations using Odefy, and default parameter values were used (tau = 1; k = 0.5; n = 3). Simulation was performed for 25-time steps (arbitrary units). Network nodes are shown on the y-axis, and the heatmap represents the activation state of each node. The activation states of nodes are continuous between 0 (dark blue) and 1 (dark red) as shown in the legend.


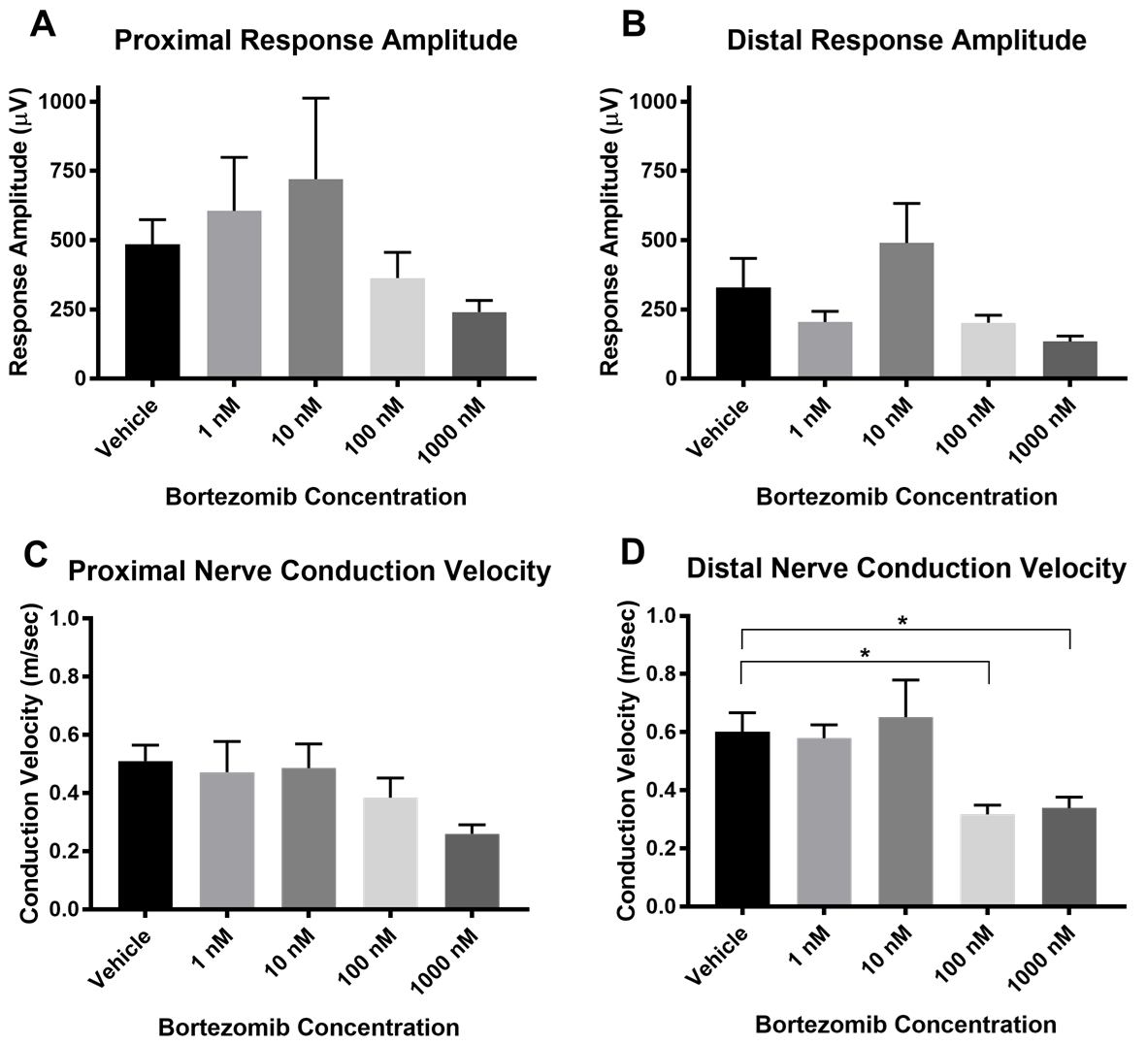


**Supplementary Figure S5.** **Bortezomib exposure-response relationship for electrophysiology endpoints using nerve-on-a-chip.** Nerve-on-a-chip (A) proximal action potential amplitude, (B) distal action potential amplitude, (C) proximal nerve conduction velocity, and (D) distal nerve conduction velocity measured across various concentrations of bortezomib (n = 4-6). GraphPad prism v7.04 was used to perform a one-way ANOVA with Dunnet’s correction for multiple comparisons. Comparisons were made for each group with respect to the vehicle control. P-values are reported as: * (<0.05), ** (<0.01), and *** (<0.001).


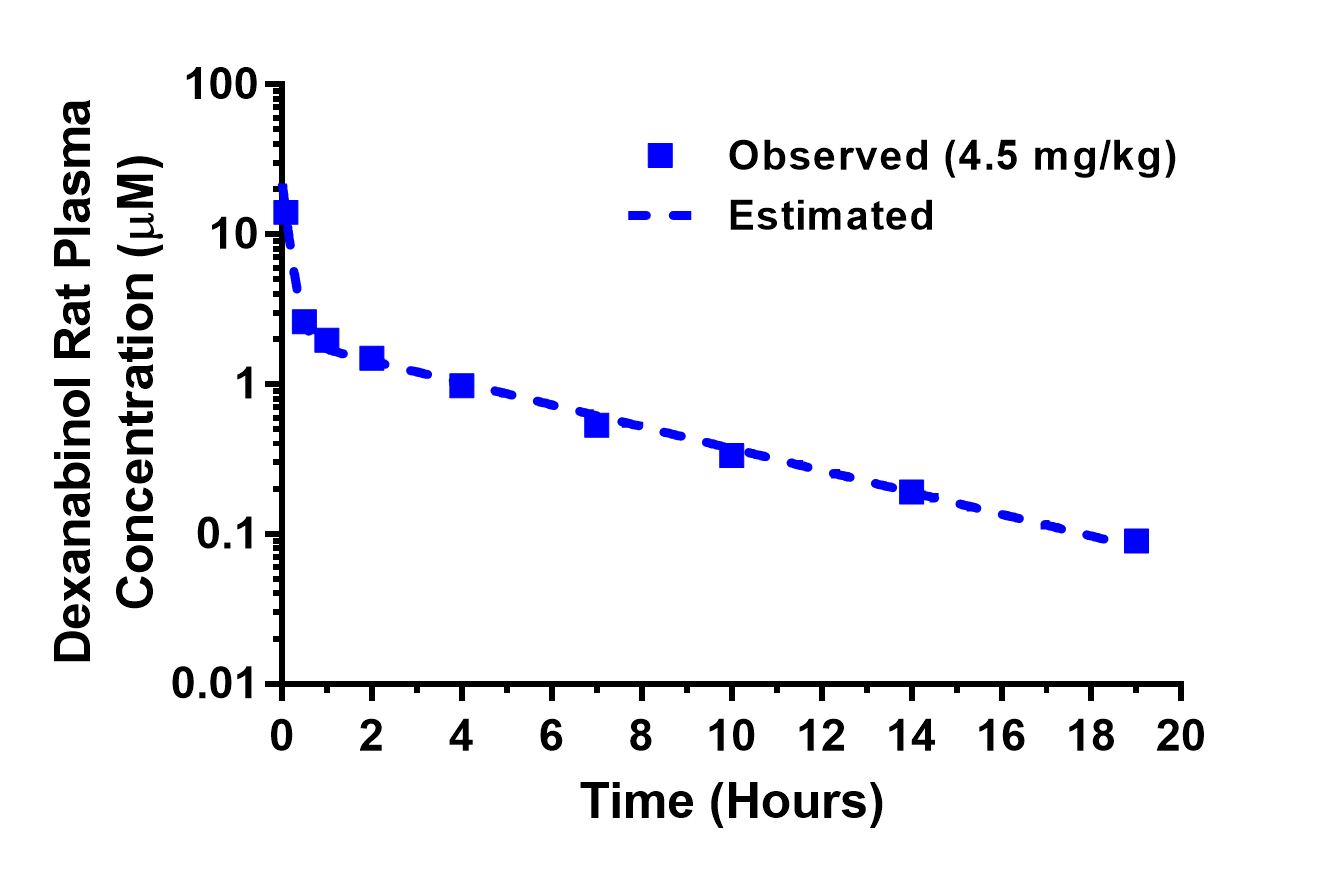


**
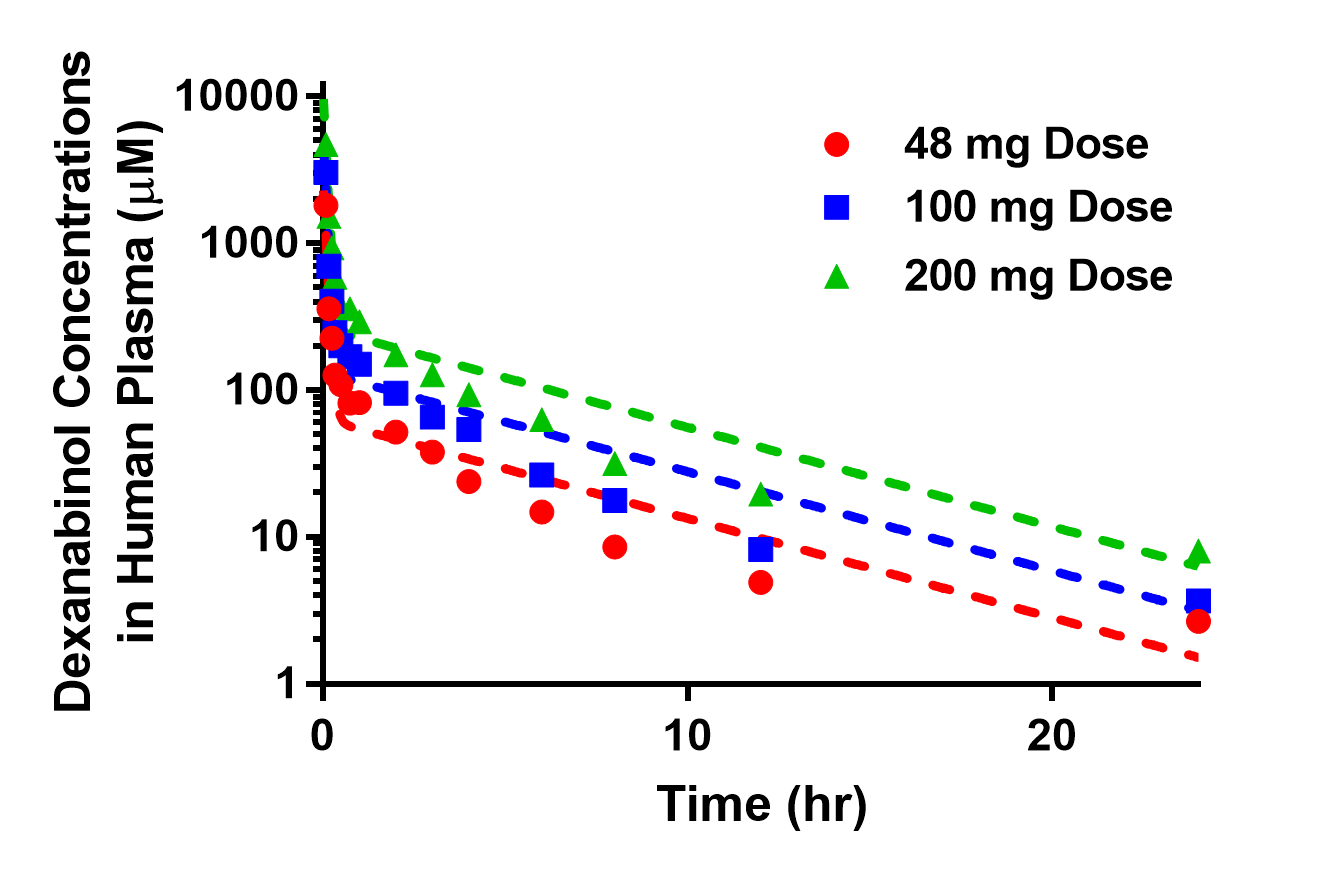
**

**Supplementary Figure S6.** **Fitted dexanabinol pharmacokinetics in rats and humans.** (Top) Pharmacokinetic model fits to plasma concentrations from rats given dexanabinol IV (4.5 mg/kg). (Bottom) Pharmacokinetic model fits to plasma concentrations from human volunteers given dexanabinol IV (48, 100, and 200 mg). Observed plasma concentrations and model fitted profiles are displayed as solid markers and dashed lines. Parameter estimates are listed in Table S6.


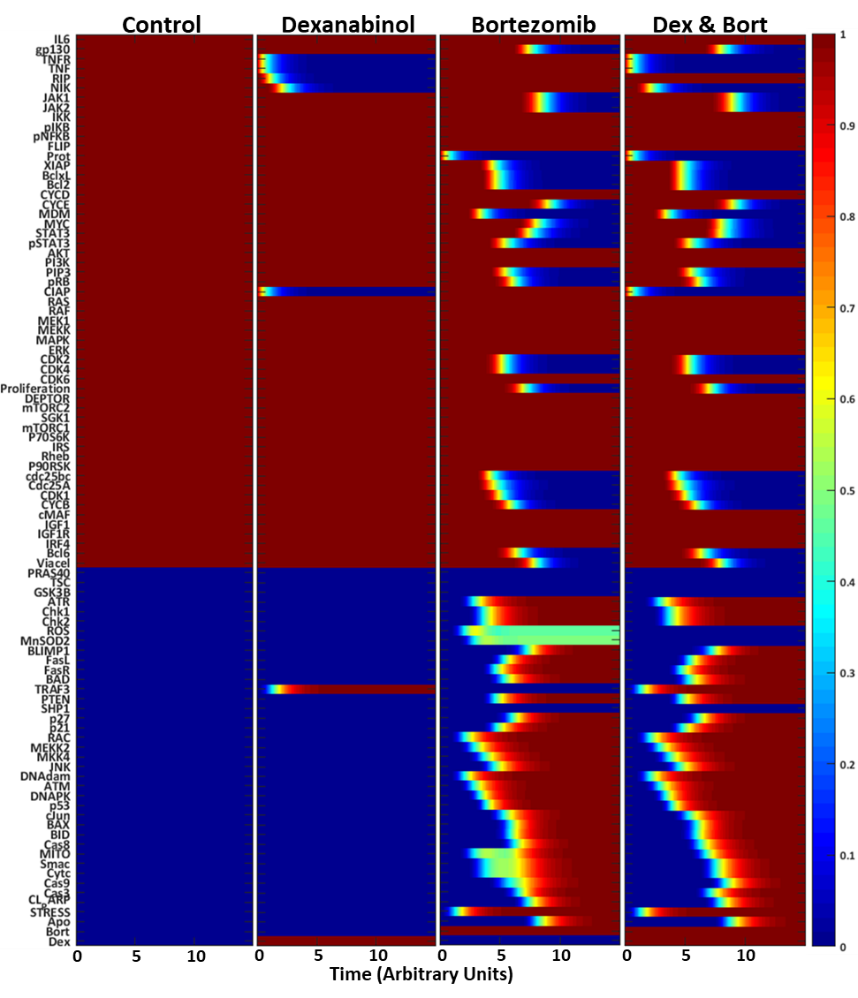


**Supplementary Figure S7.** **Multiple myeloma signaling network simulations.** Boolean network model was converted to normalized HillCube differential equations using Odefy, and default parameter values were used (tau = 1; k = 0.5; n = 3). Model predictions for the activation state of network components over time are displayed as heatmaps for control, dexanabinol monotherapy, bortezomib monotherapy, and the combination of both drugs. The key on the right-hand side of the figure indicates the magnitude of activation for network species, which ranges between zero (blue) and one (red). Model-predicted activation state dynamics of select network components are displayed for bortezomib monotherapy and its combination with dexanabinol. Proteasome (green), TNFα receptor (purple), reactive oxygen species (blue), caspase 3 (orange), cytochrome C (light blue), and apoptosis (red).

## Supplementary Tables

**Supplementary Table S1.** Systems pharmacology model equations, node initial conditions, and supporting references.

| **Node Name** | **Boolean Logic Functions** | **Initial Condition** | **References** |
| --- | --- | --- | --- |
| Akt | PIP3 \|\| !Trib3 | 1 | (Brunet et al., 2001;Zareen et al., 2013) |
| APCCdh1 | !Cdk5 | 1 | (de Tudela et al., 2015) |
| Apoptosis | cParp1 | 0 | (Chaitanya et al., 2010) |
| Arf | Myc && Foxo3 | 0 | (Zindy et al., 1998;Li et al., 2008) |
| Ask1 | Daxx \|\| Traf2 | 0 | (Nishitoh et al., 1998;Nishitoh et al., 2002) |
| Atf2 | JNK \|\| p38 | 0 | (Buschmann et al., 1998;Lindwall et al., 2004;Roberts and Der, 2007) |
| Atf3 | Atf2 \|\| Perk \|\| Atf4 | 0 | (Jiang et al., 2004;Lindwall et al., 2004;Wek et al., 2006;Lee et al., 2010) |
| Atf4 | Camk && Rsk2 \|\| Mapkapk2 \|\| Eif2s1 \|\| Jnk | 0 | (Xing et al., 1996;Harding et al., 2000;Novoa et al., 2001;Jiang et al., 2004;Proud, 2005;Wek et al., 2006;Matsuguchi et al., 2009) |
| Atf6 | ERStress \|\| !Bip | 0 | (Shen et al., 2002;Walter and Ron, 2011) |
| Atm | ROS | 0 | (Alexander et al., 2010) |
| ATP | !mPTP | 1 | (Crompton, 1999) |
| Axonal Transport | Khc && ATP \|\| Klc && ATP | 1 | (Goldstein and Yang, 2000) |
| Bad | !Akt \|\| p53 \|\| Jnk | 0 | (Datta et al., 1997;Donovan et al., 2002;Jiang et al., 2006) |
| Bax | BID \|\| !Bcl2 && !Bclxl && Myc \|\| Bim && Puma && !Bcl2 && !Bclxl | 0 | (Yang et al., 1995;Soucie et al., 2001;Juin et al., 2002;Dansen et al., 2006;Kristiansen and Ham, 2014) |
| Bcl2 | Stat \|\| !Chop && Nfkb \|\| !Bad | 1 | (Yang et al., 1995;Tamatani et al., 1999;Hirano et al., 2000;McCullough et al., 2001) |
| Bclxl | !Bad \|\| Nfkb && !Puma | 1 | (Yang et al., 1995;Tamatani et al., 1999;Follis et al., 2013;Kristiansen and Ham, 2014) |
| Bcmyb | E2f1 | 0 | (Biswas et al., 2005) |
| Bdnf | <> | 1 | - |
| Bid | Casp8 \|\| Casp10 | 0 | (Li et al., 1998;Brunet et al., 2001;Milhas et al., 2005) |
| Bim | Chop \|\| Bcmyb && cJun && Foxo3 | 0 | (Whitfield et al., 2001;Biswas et al., 2005;Ghosh et al., 2012) |
| Bip | ERStress \|\| Bip | 1 | (Bertolotti et al., 2000;Shen et al., 2002) |
| Bortezomib | <> | 0 | - |
| Calcium | Ip3r && Bip && ERStress \|\| Nmdar | 0 | (MacDermott et al., 1986;Berridge, 1993;Shen and Meyer, 1999;Pinton et al., 2008) |
| Calm | Calcium | 0 | (Crivici and Ikura, 1995;Shen and Meyer, 1999) |
| Calpain | Calcium | 0 | (Croall and DeMartino, 1991;Nakagawa and Yuan, 2000) |
| Camk | Calm | 0 | (Shen and Meyer, 1999) |
| Casp10 | Fadd | 0 | (Kischkel et al., 2001) |
| Casp12 | Calpain \|\| Casp7 | 0 | (Nakagawa and Yuan, 2000;Rao et al., 2004) |
| Casp3 | Casp9 && !Xiap \|\| Casp8 \|\| Casp10 | 0 | (Slee et al., 1999;Brunet et al., 2001;Kischkel et al., 2001;Boatright and Salvesen, 2003;Scott et al., 2005) |
| Casp7 | Casp10 \|\| Casp8 \|\| Casp9 && !Xiap | 0 | (Slee et al., 1999;Kischkel et al., 2001;Boatright and Salvesen, 2003;Scott et al., 2005) |
| Casp8 | Fadd | 0 | (Ashkenazi and Dixit, 1998;Brunet et al., 2001) |
| Casp9 | Casp12 && !Xiap && CytC | 0 | (Slee et al., 1999;Brunet et al., 2001;Srinivasula et al., 2001;Morishima et al., 2002;Rao et al., 2004) |
| Cdc42Rac1 | Ngfr | 1 | (Nusser et al., 2002;Aoki et al., 2004) |
| Cdk2 | CycE | 0 | (Sherr and Roberts, 1999) |
| Cdk4/6 | CycD | 0 | (Sherr and Roberts, 1999) |
| Cdk5 | Nmdar && Calpain | 0 | (Cheung and Ip, 2004) |
| Chop | Atf4 \|\| Xbp1 \|\| p38 \|\| Atf6 | 0 | (Wang and Ron, 1996;Harding et al., 2000;Jiang et al., 2004;Oyadomari and Mori, 2004;Wek et al., 2006;Roberts and Der, 2007) |
| cJun | Jnk | 0 | (Buschmann et al., 1998;Lindwall et al., 2004) |
| cParp1 | Parp1 | 0 | (Chaitanya et al., 2010) |
| cPkc | Calcium && Dag | 0 | (Nishizuka, 1984;Tanaka and Nishizuka, 1994) |
| Cyc | Bax && mPTP | 0 | (Crompton, 1999;Brunet et al., 2001) |
| CycD | !Gsk3b && !Pcna && !p21 && !p15 \|\| !Ngfr | 0 | (Xiong et al., 1993;Freeman et al., 1994;Sherr and Roberts, 1999) |
| CycE | CycD && Cdk4/6 && !p21 | 0 | (Xiong et al., 1993;Sherr and Roberts, 1999) |
| Dag | Plcg1 | 1 | (Nishizuka, 1984) |
| Daxx | Fasr | 0 | (Yang et al., 1997) |
| E2f1 | !pRb | 0 | (Biswas et al., 2005) |
| Eif2s1 | Perk && !Gadd34 \|\| Perk | 0 | (Harding et al., 2000;Novoa et al., 2001;Novoa et al., 2003;Jiang et al., 2004;Proud, 2005;Wek et al., 2006) |
| Erk1/2 | Mek1/2 | 1 | (Johnson and Lapadat, 2002;Roberts and Der, 2007) |
| Erk5 | Mek5 | 1 | (Johnson and Lapadat, 2002;Roberts and Der, 2007) |
| ERStress | ROS | 0 | (Kaufman, 1999;Hasnain et al., 2012;Nakka et al., 2016) |
| Fadd | FasR \|\| Tradd | 0 | (Ashkenazi and Dixit, 1998) |
| FasL | cJun && FOXO3 | 0 | (Brunet et al., 2001) |
| FasR | FasL | 0 | (Ashkenazi and Dixit, 1998) |
| Foxo3 | !Akt | 0 | (Zareen et al., 2013) |
| Gadd34 | CHOP | 0 | (Jiang et al., 2004) |
| Gadd45 | p53 | 0 | (Smith et al., 1994) |
| Grb2sos | TrkB \|\| !RSK2 | 1 | (Aronheim et al., 1994) |
| Gsk3b | !Akt \|\| Nmdar \|\| !Bdnf | 0 | (Mai et al., 2002;Sharma et al., 2002;Ortega et al., 2010) |
| Htra2Omi | !Akt && mPTP && Bax \|\| mPTP && Bax | 0 | (Yamaguchi et al., 2003;Yang et al., 2007) |
| Ikba | !Ikk \|\| Nfkb | 1 | (Brown et al., 1993;DiDonato et al., 1997) |
| Ikk | Nik && Akt | 1 | (Ozes et al., 1999) |
| Il1b | <> | 0 | - |
| Il1r | Il1b | 0 | (Dinarello, 1989) |
| Il6 | <> | 0 | - |
| Il6r | Il6 | 0 | (Kishimoto, 2006) |
| IP3 | Plcg1 | 1 | (Lemmon et al., 1995) |
| Ip3r | Ip3 | 1 | (Mignery and Südhof, 1990) |
| Ire1 | ERStress \|\| !Bip | 0 | (Bertolotti et al., 2000;Nishitoh et al., 2002) |
| Jak | Il6r | 0 | (Hirano et al., 2000;Yamauchi et al., 2006;Dominguez et al., 2008) |
| Jnk | Mkk4 && Mkk7 \|\| NO | 0 | (Kim et al., 1997;Nishitoh et al., 1998;Lisnock et al., 2000;Nishitoh et al., 2002;Roberts and Der, 2007) |
| Khc | !Jnk | 1 | (Morfini et al., 2006) |
| Klc | !Gsk3b | 1 | (Morfini et al., 2002) |
| Mapkapk2 | p38 | 0 | (Stokoe et al., 1992;Rouse et al., 1994) |
| Mdm2 | !Arf \|\| !p38 | 1 | (Pomerantz et al., 1998;Zhu et al., 2002) |
| Mek1/2 | Mekk1 \|\| Raf \|\| nPkc | 1 | (Johnson and Lapadat, 2002;Roberts and Der, 2007) |
| Mek5 | Mekk3 | 1 | (Chao et al., 1999;Roberts and Der, 2007) |
| Mekk1 | Cdc42Rac1 \|\| Traf2 | 1 | (Kaga et al., 1998;Nishitoh et al., 1998) |
| Mekk3 | Ras | 1 | (Minden et al., 1994;Davis, 1995) |
| Mkk3 | Ask1 \|\| MLK3 | 0 | (Tibbles et al., 1996;Ichijo et al., 1997;Roberts and Der, 2007) |
| Mkk4 | Ask1 && Tak1 && Mekk1 \|\| Mlk3 | 0 | (Minden et al., 1995;Tibbles et al., 1996;Ichijo et al., 1997;HuangFu et al., 2006;Roberts and Der, 2007) |
| Mkk6 | Ask1 \|\| Mlk3 | 0 | (Tibbles et al., 1996;Ichijo et al., 1997;Roberts and Der, 2007) |
| Mkk7 | Ask1 && Tak1 && Mekk1 \|\| Mlk3 | 0 | (Minden et al., 1995;Tibbles et al., 1996;Ichijo et al., 1997;HuangFu et al., 2006;Roberts and Der, 2007) |
| Mlk3 | !Akt && !Ngfr | 0 | (Xu et al., 2001;Zhang et al., 2006) |
| mPTP | Calcium \|\| Ros | 0 | (Crompton, 1999;Brustovetsky et al., 2002;Pinton et al., 2008;Tajeddine, 2016) |
| Myc | !Gsk3b \|\| Stat \|\| !Smad4 \|\| !Arf | 1 | (Qi et al., 2004;Li et al., 2008) |
| NerveDamage | Atf3 | 0 | (Obata et al., 2003) |
| Nfkb | Eif2s1 \|\| !Gsk3b \|\| !Ikba | 1 | (Brown et al., 1993;Proud, 2005;Wek et al., 2006) |
| Ngfb | <> | 1 | - |
| Ngfr | Bdnf \|\| Ngfb | 1 | (Radeke et al., 1987;Rodriguez-Tebar et al., 1990) |
| Nik | Ripk1 \|\| Mekk1 && Tak1 | 1 | (Ashkenazi and Dixit, 1998;Wong et al., 2010;Boutaffala et al., 2015) |
| Nmda | <> | 0 | - |
| Nmdar | Nmda | 0 | (Moriyoshi et al., 1991) |
| nNos | Nmdar | 0 | (Girouard et al., 2009) |
| NO | nNos | 0 | (Girouard et al., 2009) |
| Nox2 | Nmdar && Atf4 && cPkc | 0 | (Girouard et al., 2009;Raad et al., 2009;Srivastava et al., 2016) |
| nPkc | Dag | 1 | (Nishizuka, 1984;Tanaka and Nishizuka, 1994) |
| p15 | !Myc | 0 | (Staller et al., 2001) |
| p21 | !Akt \|\| p53 \|\| Stat \|\| APCCdh1 | 1 | (Hirano et al., 2000;Rössig et al., 2001;Zhao et al., 2003;Almeida, 2012) |
| p38 | Mkk3 && Ras && Mkk6 | 0 | (Raingeaud et al., 1996;Roberts and Der, 2007) |
| p53 | !Mdm2 \|\| Jnk && Atm && p38 | 0 | (Qi et al., 2004;Roberts and Der, 2007;Li et al., 2008) |
| Parp1 | Casp3 \|\| Casp7 | 0 | (Chaitanya et al., 2010) |
| Pcna | Gadd45 | 0 | (Smith et al., 1994) |
| Perk | ERStress \|\| !Bip | 0 | (Bertolotti et al., 2000;Novoa et al., 2001;Jiang et al., 2004;Walter and Ron, 2011) |
| Pi3k | Trka \|\| Jak \|\| Trkb \|\| Ras | 1 | (Brunet et al., 2001;Rawlings et al., 2004;Castellano and Downward, 2011;Kristiansen and Ham, 2014) |
| Pip3 | Pi3k \|\| !Pten | 1 | (Brunet et al., 2001;Wee et al., 2009) |
| Plcg1 | Trkb | 1 | (Schlessinger and Ullrich, 1992) |
| pRb | !CycD && !Cdk46 \|\| !Cdk2 && !CycE | 1 | (Weinberg, 1995;Sherr and Roberts, 1999) |
| Proliferation | !pRb | 0 | (Weinberg, 1995) |
| Proteasome | <> | 1 | - |
| Pten | p53 | 0 | (Stambolic et al., 2001;Wee et al., 2009) |
| PUMA | p53 && Chop && Nfkb | 0 | (Nakano and Vousden, 2001;Wang et al., 2009;Galehdar et al., 2010) |
| Raf | cPkc \|\| Ras | 1 | (Marais et al., 1998;Kristiansen and Ham, 2014) |
| Ras | !Erk1_2 \|\| Jak \|\| Calcium \|\|Grb2sos | 1 | (Aronheim et al., 1994;Farnsworth et al., 1995;Kolch, 2000;Rawlings et al., 2004) |
| Ripk1 | Tradd | 0 | (Ashkenazi and Dixit, 1998) |
| ROS | Chop \|\| !Proteasome \|\| Nox2 && NO | 0 | (Ding and Keller, 2001;McCullough et al., 2001;Marciniak et al., 2004;Girouard et al., 2009;Choi et al., 2016) |
| Rsk2 | Erk1/2 \|\| Erk5 | 1 | (Cargnello and Roux, 2011) |
| Smad2/3 | Tgfbr | 0 | (Heldin et al., 1997) |
| Smad4 | Smad2_3 \|\| Jnk | 0 | (Heldin et al., 1997;Engel et al., 1999) |
| Stat | Jak | 0 | (Hirano et al., 2000;Yamauchi et al., 2006;Dominguez et al., 2008) |
| Tak1 | Traf2 \|\| Traf6 \|\| !Xiap | 1 | (Yamaguchi et al., 1999;Chen et al., 2006;Landström, 2010) |
| Tgfb | <> | 0 | - |
| Tgfbr | Tgfb | 0 | (Massagué, 1998) |
| Tnfa | <> | 0 | - |
| Tnfr | Tnfa | 0 | (Ashkenazi and Dixit, 1998;Nishitoh et al., 1998) |
| Tradd | Tnfr | 0 | (Ashkenazi and Dixit, 1998;Nishitoh et al., 1998) |
| Traf2 | Ire1 \|\| Tradd \|\| Il1r | 0 | (Ashkenazi and Dixit, 1998;Nishitoh et al., 1998;Nishitoh et al., 2002) |
| Traf6 | Ngfr \|\| Tgfbr \|\| Il1r | 1 | (Cao et al., 1996;Khursigara et al., 1999;Yamashita et al., 2008) |
| Trkb | Bdnf | 1 | (Brunet et al., 2001) |
| Trib3 | !Ngfr && Foxo3 \|\| Atf4 && Chop | 0 | (Ohoka et al., 2005;Zareen et al., 2013) |
| Trka | Ngfb | 1 | (Brunet et al., 2001;Nusser et al., 2002;Kristiansen and Ham, 2014) |
| Xbp1 | Ire1 | 0 | (Plongthongkum et al., 2007) |
| Xiap | Nfkb && !Htra2Omi | 1 | (Stehlik et al., 1998;Suzuki et al., 2001) |

**Supplementary Table S2.** Parameter Estimates from Bortezomib and Dexanabinol Cytotoxicity Data in U266 cells after 24 hours of exposure.

| **Parameters** | **Units** | **Value** | **CV (%)** | **Confidence Interval (95%)** |
| --- | --- | --- | --- | --- |
| **IC_50D_** | μM | 20.7 | 9.9 | [ 16.4 - 25.1 ] |
| **I_maxD_** | - | 0.951 | 3.6 | [ 0.878 - 1.02 ] |
| **γ_D_** | - | 5.00 | Fixed | Fixed |
| **IC_50B_** | nM | 1.07 | 28.5 | [ 0.853 - 0.907 ] |
| **I_maxB_** | - | 0.880 | 1.5 | [ 0.429 - 1.70 ] |
| **γ_B_** | - | 1.00 | Fixed | Fixed |
| **E_0_** | % | 100 | Fixed | Fixed |
| **Ψ** | - | 2.71 | 21.8 | [ 1.53 - 3.89 ] |

**Supplementary Table S3.** Parameter estimates of bortezomib and dexanabinol cytotoxicity in U266 cells after 48 hours of exposure.

| **Parameters** | **Units** | **Value** | **CV (%)** | **Confidence Interval (95%)** |
| --- | --- | --- | --- | --- |
| **IC_50D_** | μM | 19.5 | 16.6 | [ 12.7 - 26.4 ] |
| **I_maxD_** | - | 0.990 | 4.9 | [ 0.887 - 1.09 ] |
| **γ_D_** | - | 5.00 | Fixed | Fixed |
| **IC_50B_** | nM | 1.84 | 21.5 | [ 1.01 - 2.66 ] |
| **I_maxB_** | - | 1.00 | Fixed | Fixed |
| **γ_B_** | - | 0.998 | 0.8 | [ 0.980 - 1.02 ] |
| **E_0_** | % | 100 | - | Fixed |
| **Ψ** | - | 2.10 | 26.5 | [ 0.985 - 3.21 ] |

**Supplementary Table S4.** Parameter estimates of bortezomib and dexanabinol cytotoxicity in U266 cells after 72 hours of exposure.

| **Parameters** | **Units** | **Value** | **CV (%)** | **Confidence Interval (95%)** |
| --- | --- | --- | --- | --- |
| **IC_50D_** | μM | 18.0 | 8.0 | [ 14.9 - 21.0] |
| **I_maxD_** | - | 1.00 | Fixed | Fixed |
| **γ_D_** | - | 5.00 | Fixed | Fixed |
| **IC_50_B** | nM | 2.10 | 25.0 | [ 1.00 - 3.19 ] |
| **I_maxB_** | - | 1.00 | Fixed | Fixed |
| **γ_B_** | - | 1.00 | 0.9 | [ 0.983 - 1.02 ] |
| **E_0_** | % | 100 | - | Fixed |
| **ψ** | - | 1.40 | 17.2 | [ 0.919 - 1.88 ] |

**Supplementary Table S5.** Bortezomib Pharmacokinetic Parameters in Mice.

| **Parameters** | **Units** | **Value** | **CV (%)** |
| --- | --- | --- | --- |
| **V1** | L | 0.0220 | 28.9 |
| **CL** | L/hr | 0.00434 | 21.0 |
| **K_12_** | 1/hr | 12.4 | 18.7 |
| **K_21_** | 1/hr | 0.297 | 17.5 |
| **Rmax** | nM | 561 | 34.6 |
| **Kd** | nM | 0.562 | 70.9 |
| **Σ** | - | 0.213 | 23.9 |

**Supplementary Table S6.** Pharmacokinetic model parameters and allometric scaling of dexanabinol.

|  | **Species:** | **Rat** | | **Human** | | **(Interspecies)** | | **Mouse** |
| --- | --- | --- | --- | --- | --- | --- | --- | --- |
| **Parameters** | **Units** | **Estimated** | **CV (%)** | **Estimated** | **CV (%)** | **Beta** | **Alpha** | **Predicted** |
| **V** | L | 0.207 | 11.9 | 15.6 | 28.0 | 0.82 | 0.44 | 0.0162 |
| **CL** | L/hr | 0.308 | 3.0 | 73.3 | 8.4 | 1.04 | 0.80 | 0.0122 |
| **VT** | L | 1.25 | 5.0 | 280 | 14.7 | 1.03 | 3.21 | 0.0514 |
| **CLD** | L/hr | 0.913 | 11.1 | 113 | 23.6 | 0.92 | 2.11 | 0.0532 |
| **Σ** | - | 0.084 | 23.7 | 0.377 | 12.4 | - | - | - |
| **BW (kg)** | kg | 0.400 | - | 77.4 | - | - | - | 0.0179 |
| **Half-life** | hr | 4.13 | - | 4.44 | - | - | - | 10.8 |

**Supplementary Table S7.** Pharmacodynamic parameters of bortezomib and dexanabinol effects on tumor volume in SCID mice bearing MM1.S multiple myeloma xenografts.

| **Parameters** | **Units** | **Value** | **CV (%)** | **Confidence Interval (95%)** |
| --- | --- | --- | --- | --- |
| **kgr** | 1/hr | 1.57E-03 | 26.5 | [0.707E-03, 0.244E-02] |
| **Kdex** | 1/(hr⋅μM) | 5.42E-04 | Fixed | Fixed |
| **Kbort** | 1/(hr⋅nM) | 2.82E-04 | 24.7 | [0.139E-03, 0.427E-03] |
| **kres** | 1/hr | 7.60E-03 | 21.3 | [0.423E-02, 0.110E-01] |
| **ψ** | - | 0.883 | 4.7 | [0.796 , 0.970] |
| **kg_0_** | 1/hr | 9.82E-03 | 6.8 | [0.844E-02, 0.112E-01] |
| **σ** | - | 0.143 | 13.8 | [ 0.102 , 0.185] |

**Supplementary Table S8.** Dexanabinol *in vitro* pharmacodynamic properties

| **Reference** | **Experimental Model** | **Pharmacodynamic Effect** | **Experimental Biomarker** | **EC_50_, IC_50_ (μM)** |
| --- | --- | --- | --- | --- |
| **(Shohami et al., 1997)** | Macrophages (RAW 264.7) | TNFα Inhibition | LPS induced TNFα production | 9.1 |
| **(Eshhar et al., 1993)** | Rat-derived Cortical Neurons | NMDA antagonism | NMDA-induced neurotoxicity (XTT) | 3.6 |
| **(Nadler et al., 1993b)** | Rat-derived Cortical Neurons | NMDA antagonism | LDH release | 9.0 |
| **(Nadler et al., 1993a)** | Rat-derived Cortical Neurons | NMDA antagonism | ^45^Ca^2+^ Uptake | 30.4 |
| **(Eshhar et al., 1995)** | Methyl Linoleate Oxidation | Antioxidant | Oxygen Consumption | 29.7 |

# Supplementary References

Alexander, A., Cai, S.-L., Kim, J., Nanez, A., Sahin, M., Maclean, K.H., Inoki, K., Guan, K.-L., Shen, J., and Person, M.D. (2010). ATM signals to TSC2 in the cytoplasm to regulate mTORC1 in response to ROS. *Proceedings of the National Academy of Sciences* 107**,** 4153-4158.

Almeida, A. (2012). Regulation of APC/C-Cdh1 and its function in neuronal survival. *Molecular neurobiology* 46**,** 547-554.

Aoki, K., Nakamura, T., and Matsuda, M. (2004). Spatio-temporal regulation of Rac1 and Cdc42 activity during nerve growth factor-induced neurite outgrowth in PC12 cells. *Journal of Biological Chemistry* 279**,** 713-719.

Aronheim, A., Engelberg, D., Li, N., Al-Alawi, N., Schlessinger, J., and Karin, M. (1994). Membrane targeting of the nucleotide exchange factor Sos is sufficient for activating the Ras signaling pathway. *Cell* 78**,** 949-961.

Ashkenazi, A., and Dixit, V.M. (1998). Death receptors: signaling and modulation. *Science***,** 1305-1308.

Berridge, M.J. (1993). Inositol trisphosphate and calcium signalling. *Nature* 361**,** 315.

Bertolotti, A., Zhang, Y., Hendershot, L.M., Harding, H.P., and Ron, D. (2000). Dynamic interaction of BiP and ER stress transducers in the unfolded-protein response. *Nature cell biology* 2**,** 326.

Biswas, S.C., Liu, D.X., and Greene, L.A. (2005). Bim is a direct target of a neuronal E2F-dependent apoptotic pathway. *Journal of Neuroscience* 25**,** 8349-8358.

Boatright, K.M., and Salvesen, G.S. (2003). Mechanisms of caspase activation. *Current opinion in cell biology* 15**,** 725-731.

Boutaffala, L., Bertrand, M., Remouchamps, C., Seleznik, G., Reisinger, F., Janas, M., Bénézech, C., Fernandes, M., Marchetti, S., and Mair, F. (2015). NIK promotes tissue destruction independently of the alternative NF-κB pathway through TNFR1/RIP1-induced apoptosis. *Cell death and differentiation* 22**,** 2020.

Brown, K., Park, S., Kanno, T., Franzoso, G., and Siebenlist, U. (1993). Mutual regulation of the transcriptional activator NF-kappa B and its inhibitor, I kappa B-alpha. *Proceedings of the National Academy of Sciences* 90**,** 2532-2536.

Brunet, A., Datta, S.R., and Greenberg, M.E. (2001). Transcription-dependent and-independent control of neuronal survival by the PI3K–Akt signaling pathway. *Current opinion in neurobiology* 11**,** 297-305.

Brustovetsky, N., Brustovetsky, T., Jemmerson, R., and Dubinsky, J.M. (2002). Calcium‐induced Cytochrome c release from CNS mitochondria is associated with the permeability transition and rupture of the outer membrane. *Journal of neurochemistry* 80**,** 207-218.

Buschmann, T., Martin-Villalba, A., Kocsis, J., Waxman, S., Zimmermann, M., and Herdegen, T. (1998). Expression of Jun, Fos and ATF-2 proteins in axotomized explanted and cultured adult rat dorsal root ganglia. *Neuroscience* 84**,** 163-176.

Cao, Z., Xiong, J., Takeuchi, M., Kurama, T., and Goeddel, D.V. (1996). TRAF6 is a signal transducer for interleukin-1. *Nature* 383**,** 443.

Cargnello, M., and Roux, P.P. (2011). Activation and function of the MAPKs and their substrates, the MAPK-activated protein kinases. *Microbiology and molecular biology reviews* 75**,** 50-83.

Castellano, E., and Downward, J. (2011). RAS interaction with PI3K: more than just another effector pathway. *Genes & cancer* 2**,** 261-274.

Chaitanya, G.V., Alexander, J.S., and Babu, P.P. (2010). PARP-1 cleavage fragments: signatures of cell-death proteases in neurodegeneration. *Cell Communication and Signaling* 8**,** 31.

Chao, T.-H., Hayashi, M., Tapping, R.I., Kato, Y., and Lee, J.-D. (1999). MEKK3 directly regulates MEK5 activity as part of the big mitogen-activated protein kinase 1 (BMK1) signaling pathway. *Journal of Biological Chemistry* 274**,** 36035-36038.

Chen, Z., Bhoj, V., and Seth, R. (2006). "Ubiquitin, TAK1 and IKK: is there a connection?". Nature Publishing Group).

Cheung, Z.H., and Ip, N.Y. (2004). Cdk5: mediator of neuronal death and survival. *Neuroscience letters* 361**,** 47-51.

Choi, S.-R., Kwon, S.-G., Choi, H.-S., Han, H.-J., Beitz, A.J., and Lee, J.-H. (2016). Neuronal NOS activates spinal NADPH oxidase 2 contributing to central sigma-1 receptor-induced pain hypersensitivity in mice. *Biological and Pharmaceutical Bulletin* 39**,** 1922-1931.

Crivici, A., and Ikura, M. (1995). Molecular and structural basis of target recognition by calmodulin. *Annual review of biophysics and biomolecular structure* 24**,** 85-116.

Croall, D.E., and Demartino, G.N. (1991). Calcium-activated neutral protease (calpain) system: structure, function, and regulation. *Physiological reviews* 71**,** 813-847.

Crompton, M. (1999). The mitochondrial permeability transition pore and its role in cell death. *Biochemical Journal* 341**,** 233-249.

Dansen, T.B., Whitfield, J., Rostker, F., Brown-Swigart, L., and Evan, G.I. (2006). Specific requirement for Bax, not Bak, in Myc-induced apoptosis and tumor suppression in vivo. *Journal of Biological Chemistry* 281**,** 10890-10895.

Datta, S.R., Dudek, H., Tao, X., Masters, S., Fu, H., Gotoh, Y., and Greenberg, M.E. (1997). Akt phosphorylation of BAD couples survival signals to the cell-intrinsic death machinery. *Cell* 91**,** 231-241.

Davis, R.J. (1995). Transcriptional regulation by MAP kinases. *Mol Reprod Dev* 42**,** 459-467.

De Tudela, M.V.-P., Maestre, C., Delgado-Esteban, M., Bolaños, J.P., and Almeida, A. (2015). Cdk5-mediated inhibition of APC/C-Cdh1 switches on the cyclin D1-Cdk4-pRb pathway causing aberrant S-phase entry of postmitotic neurons. *Scientific reports* 5**,** 18180.

Didonato, J.A., Hayakawa, M., Rothwarf, D.M., Zandi, E., and Karin, M. (1997). A cytokine-responsive IκB kinase that activates the transcription factor NF-κB. *Nature* 388**,** 548.

Dinarello, C.A. (1989). "Interleukin-1 and its biologically related cytokines," in *Advances in immunology*. Elsevier), 153-205.

Ding, Q., and Keller, J.N. (2001). Proteasome inhibition in oxidative stress neurotoxicity: implications for heat shock proteins. *Journal of neurochemistry* 77**,** 1010-1017.

Dominguez, E., Rivat, C., Pommier, B., Mauborgne, A., and Pohl, M. (2008). JAK/STAT3 pathway is activated in spinal cord microglia after peripheral nerve injury and contributes to neuropathic pain development in rat. *Journal of neurochemistry* 107**,** 50-60.

Donovan, N., Becker, E.B., Konishi, Y., and Bonni, A. (2002). JNK phosphorylation and activation of BAD couples the stress-activated signaling pathway to the cell death machinery. *Journal of Biological Chemistry* 277**,** 40944-40949.

Engel, M.E., Mcdonnell, M.A., Law, B.K., and Moses, H.L. (1999). Interdependent SMAD and JNK signaling in transforming growth factor-β-mediated transcription. *Journal of Biological Chemistry* 274**,** 37413-37420.

Eshhar, N., Striem, S., and Biegon, A. (1993). HU-211, a non-psychotropic cannabinoid, rescues cortical neurones from excitatory amino acid toxicity in culture. *Neuroreport* 5**,** 237-240.

Eshhar, N., Striem, S., Kohen, R., Tirosh, O., and Biegon, A. (1995). Neuroprotective and antioxidant activities of HU-211, a novel NMDA receptor antagonist. *European journal of pharmacology* 283**,** 19-29.

Farnsworth, C.L., Freshney, N.W., Rosen, L.B., Ghosh, A., Greenberg, M.E., and Feig, L.A. (1995). Calcium activation of Ras mediated by neuronal exchange factor Ras-GRF. *Nature* 376**,** 524.

Follis, A.V., Chipuk, J.E., Fisher, J.C., Yun, M.-K., Grace, C.R., Nourse, A., Baran, K., Ou, L., Min, L., and White, S.W. (2013). PUMA binding induces partial unfolding within BCL-xL to disrupt p53 binding and promote apoptosis. *Nature chemical biology* 9**,** 163.

Freeman, R.S., Estus, S., and Johnson Jr, E.M. (1994). Analysis of cell cycle-related gene expression in postmitotic neurons: selective induction of Cyclin D1 during programmed cell death. *Neuron* 12**,** 343-355.

Galehdar, Z., Swan, P., Fuerth, B., Callaghan, S.M., Park, D.S., and Cregan, S.P. (2010). Neuronal apoptosis induced by endoplasmic reticulum stress is regulated by ATF4–CHOP-mediated induction of the Bcl-2 homology 3-only member PUMA. *Journal of Neuroscience* 30**,** 16938-16948.

Ghosh, A.P., Klocke, B.J., Ballestas, M.E., and Roth, K.A. (2012). CHOP potentially co-operates with FOXO3a in neuronal cells to regulate PUMA and BIM expression in response to ER stress. *PLoS One* 7**,** e39586.

Girouard, H., Wang, G., Gallo, E.F., Anrather, J., Zhou, P., Pickel, V.M., and Iadecola, C. (2009). NMDA receptor activation increases free radical production through nitric oxide and NOX2. *Journal of Neuroscience* 29**,** 2545-2552.

Goldstein, L.S., and Yang, Z. (2000). Microtubule-based transport systems in neurons: the roles of kinesins and dyneins. *Annual review of neuroscience* 23**,** 39-71.

Harding, H.P., Novoa, I., Zhang, Y., Zeng, H., Wek, R., Schapira, M., and Ron, D. (2000). Regulated translation initiation controls stress-induced gene expression in mammalian cells. *Molecular cell* 6**,** 1099-1108.

Hasnain, S.Z., Lourie, R., Das, I., Chen, A.C.H., and Mcguckin, M.A. (2012). The interplay between endoplasmic reticulum stress and inflammation. *Immunology and cell biology* 90**,** 260-270.

Heldin, C.-H., Miyazono, K., and Ten Dijke, P. (1997). TGF-β signalling from cell membrane to nucleus through SMAD proteins. *Nature* 390**,** 465.

Hirano, T., Ishihara, K., and Hibi, M. (2000). Roles of STAT3 in mediating the cell growth, differentiation and survival signals relayed through the IL-6 family of cytokine receptors. *Oncogene* 19**,** 2548.

Huangfu, W.-C., Omori, E., Akira, S., Matsumoto, K., and Ninomiya-Tsuji, J. (2006). Osmotic Stress Activates the TAK1-JNK Pathway While Blocking TAK1-mediated NF-κB Activation TAO2 REGULATES TAK1 PATHWAYS. *Journal of Biological Chemistry* 281**,** 28802-28810.

Ichijo, H., Nishida, E., Irie, K., Ten Dijke, P., Saitoh, M., Moriguchi, T., Takagi, M., Matsumoto, K., Miyazono, K., and Gotoh, Y. (1997). Induction of apoptosis by ASK1, a mammalian MAPKKK that activates SAPK/JNK and p38 signaling pathways. *Science* 275**,** 90-94.

Jiang, H.-Y., Wek, S.A., Mcgrath, B.C., Lu, D., Hai, T., Harding, H.P., Wang, X., Ron, D., Cavener, D.R., and Wek, R.C. (2004). Activating transcription factor 3 is integral to the eukaryotic initiation factor 2 kinase stress response. *Molecular and cellular biology* 24**,** 1365-1377.

Jiang, P., Du, W., Heese, K., and Wu, M. (2006). The Bad guy cooperates with good cop p53: Bad is transcriptionally up-regulated by p53 and forms a Bad/p53 complex at the mitochondria to induce apoptosis. *Molecular and cellular biology* 26**,** 9071-9082.

Johnson, G.L., and Lapadat, R. (2002). Mitogen-activated protein kinase pathways mediated by ERK, JNK, and p38 protein kinases. *Science* 298**,** 1911-1912.

Juin, P., Hunt, A., Littlewood, T., Griffiths, B., Swigart, L.B., Korsmeyer, S., and Evan, G. (2002). c-Myc functionally cooperates with Bax to induce apoptosis. *Molecular and cellular biology* 22**,** 6158-6169.

Kaga, S., Ragg, S., Rogers, K.A., and Ochi, A. (1998). Activation of p21-CDC42/Rac-activated kinases by CD28 signaling: p21-activated kinase (PAK) and MEK kinase 1 (MEKK1) may mediate the interplay between CD3 and CD28 signals. *The Journal of Immunology* 160**,** 4182-4189.

Kaufman, R.J. (1999). Stress signaling from the lumen of the endoplasmic reticulum: coordination of gene transcriptional and translational controls. *Genes & development* 13**,** 1211-1233.

Khursigara, G., Orlinick, J.R., and Chao, M.V. (1999). Association of the p75 neurotrophin receptor with TRAF6. *Journal of Biological Chemistry* 274**,** 2597-2600.

Kim, H., Shim, J., Han, P.-L., and Choi, E.-J. (1997). Nitric oxide modulates the c-Jun N-terminal kinase/stress-activated protein kinase activity through activating c-Jun N-terminal kinase kinase. *Biochemistry* 36**,** 13677-13681.

Kischkel, F.C., Lawrence, D.A., Tinel, A., Leblanc, H., Virmani, A., Schow, P., Gazdar, A., Blenis, J., Arnott, D., and Ashkenazi, A. (2001). Death receptor recruitment of endogenous caspase-10 and apoptosis initiation in the absence of caspase-8. *Journal of Biological Chemistry*.

Kishimoto, T. (2006). Interleukin-6: discovery of a pleiotropic cytokine. *Arthritis research & therapy* 8**,** S2.

Kolch, W. (2000). Meaningful relationships: the regulation of the Ras/Raf/MEK/ERK pathway by protein interactions. *Biochemical Journal* 351**,** 289-305.

Kristiansen, M., and Ham, J. (2014). Programmed cell death during neuronal development: the sympathetic neuron model. *Cell death and differentiation* 21**,** 1025.

Landström, M. (2010). The TAK1–TRAF6 signalling pathway. *The international journal of biochemistry & cell biology* 42**,** 585-589.

Lee, S.-H., Bahn, J.H., Whitlock, N.C., and Baek, S.J. (2010). Activating transcription factor 2 (ATF2) controls tolfenamic acid-induced ATF3 expression via MAP kinase pathways. *Oncogene* 29**,** 5182.

Lemmon, M.A., Ferguson, K.M., O'brien, R., Sigler, P.B., and Schlessinger, J. (1995). Specific and high-affinity binding of inositol phosphates to an isolated pleckstrin homology domain. *Proceedings of the National Academy of Sciences* 92**,** 10472-10476.

Li, H., Zhu, H., Xu, C.-J., and Yuan, J. (1998). Cleavage of BID by caspase 8 mediates the mitochondrial damage in the Fas pathway of apoptosis. *Cell* 94**,** 491-501.

Li, Z., Boone, D., and Hann, S.R. (2008). Nucleophosmin interacts directly with c-Myc and controls c-Myc-induced hyperproliferation and transformation. *Proceedings of the National Academy of Sciences* 105**,** 18794-18799.

Lindwall, C., Dahlin, L., Lundborg, G., and Kanje, M. (2004). Inhibition of c-Jun phosphorylation reduces axonal outgrowth of adult rat nodose ganglia and dorsal root ganglia sensory neurons. *Molecular and Cellular Neuroscience* 27**,** 267-279.

Lisnock, J., Griffin, P., Calaycay, J., Frantz, B., Parsons, J., O'keefe, S.J., and Lograsso, P. (2000). Activation of JNK3α1 requires both MKK4 and MKK7: kinetic characterization of in vitro phosphorylated JNK3α1. *Biochemistry* 39**,** 3141-3148.

Macdermott, A.B., Mayer, M.L., Westbrook, G.L., Smith, S.J., and Barker, J.L. (1986). NMDA-receptor activation increases cytoplasmic calcium concentration in cultured spinal cord neurones. *Nature* 321**,** 519.

Mai, L., Jope, R.S., and Li, X. (2002). BDNF‐mediated signal transduction is modulated by GSK3β and mood stabilizing agents. *Journal of neurochemistry* 82**,** 75-83.

Marais, R., Light, Y., Mason, C., Paterson, H., Olson, M.F., and Marshall, C.J. (1998). Requirement of Ras-GTP-Raf complexes for activation of Raf-1 by protein kinase C. *Science* 280**,** 109-112.

Marciniak, S.J., Yun, C.Y., Oyadomari, S., Novoa, I., Zhang, Y., Jungreis, R., Nagata, K., Harding, H.P., and Ron, D. (2004). CHOP induces death by promoting protein synthesis and oxidation in the stressed endoplasmic reticulum. *Genes & development* 18**,** 3066-3077.

Massagué, J. (1998). "TGF-β signal transduction". Annual Reviews 4139 El Camino Way, PO Box 10139, Palo Alto, CA 94303-0139, USA).

Matsuguchi, T., Chiba, N., Bandow, K., Kakimoto, K., Masuda, A., and Ohnishi, T. (2009). JNK activity is essential for Atf4 expression and late‐stage osteoblast differentiation. *Journal of Bone and Mineral Research* 24**,** 398-410.

Mccullough, K.D., Martindale, J.L., Klotz, L.-O., Aw, T.-Y., and Holbrook, N.J. (2001). Gadd153 sensitizes cells to endoplasmic reticulum stress by down-regulating Bcl2 and perturbing the cellular redox state. *Molecular and cellular biology* 21**,** 1249-1259.

Mignery, G.A., and Südhof, T. (1990). The ligand binding site and transduction mechanism in the inositol‐1, 4, 5‐triphosphate receptor. *The EMBO Journal* 9**,** 3893-3898.

Milhas, D., Cuvillier, O., Therville, N., Clavé, P., Thomsen, M., Levade, T., Benoist, H., and Ségui, B. (2005). Caspase-10 triggers Bid cleavage and caspase cascade activation in FasL-induced apoptosis. *Journal of Biological Chemistry*.

Minden, A., Lin, A., Claret, F.-X., Abo, A., and Karin, M. (1995). Selective activation of the JNK signaling cascadeand c-Jun transcriptional activity by the small GTPases Rac and Cdc42Hs. *Cell* 81**,** 1147-1157.

Minden, A., Lin, A., Mcmahon, M., Lange-Carter, C., Derijard, B., Davis, R.J., Johnson, G.L., and Karin, M. (1994). Differential activation of ERK and JNK mitogen-activated protein kinases by Raf-1 and MEKK. *Science* 266**,** 1719-1723.

Morfini, G., Pigino, G., Szebenyi, G., You, Y., Pollema, S., and Brady, S.T. (2006). JNK mediates pathogenic effects of polyglutamine-expanded androgen receptor on fast axonal transport. *Nature neuroscience* 9**,** 907.

Morfini, G., Szebenyi, G., Elluru, R., Ratner, N., and Brady, S.T. (2002). Glycogen synthase kinase 3 phosphorylates kinesin light chains and negatively regulates kinesin‐based motility. *The EMBO journal* 21**,** 281-293.

Morishima, N., Nakanishi, K., Takenouchi, H., Shibata, T., and Yasuhiko, Y. (2002). An ER stress-specific caspase cascade in apoptosis: cytochrome c-independent activation of caspase-9 by caspase-12. *Journal of Biological Chemistry*.

Moriyoshi, K., Masu, M., Ishii, T., Shigemoto, R., Mizuno, N., and Nakanishi, S. (1991). Molecular cloning and characterization of the rat NMDA receptor. *Nature* 354**,** 31.

Nadler, V., Mechoulam, R., and Sokolovsky, M. (1993a). Blockade of45Ca2+ influx through theN-methyl-d-aspartate receptor ion channel by the non-psychoactive cannabinoid HU-211. *Brain research* 622**,** 79-85.

Nadler, V., Mechoulam, R., and Sokolovsky, M. (1993b). The non-psychotropic cannabinoid (+)-(3S, 4S)-7-hydroxy-Δ6-tetrahydrocannabinol 1, 1-dimethylheptyl (HU-211) attenuates N-methyl-d-aspartate receptor-mediated neurotoxicity in primary cultures of rat forebrain. *Neuroscience letters* 162**,** 43-45.

Nakagawa, T., and Yuan, J. (2000). Cross-talk between two cysteine protease families: activation of caspase-12 by calpain in apoptosis. *The Journal of cell biology* 150**,** 887-894.

Nakano, K., and Vousden, K.H. (2001). PUMA, a novel proapoptotic gene, is induced by p53. *Molecular cell* 7**,** 683-694.

Nakka, V.P., Prakash-Babu, P., and Vemuganti, R. (2016). Crosstalk between endoplasmic reticulum stress, oxidative stress, and autophagy: potential therapeutic targets for acute CNS injuries. *Molecular neurobiology* 53**,** 532-544.

Nishitoh, H., Matsuzawa, A., Tobiume, K., Saegusa, K., Takeda, K., Inoue, K., Hori, S., Kakizuka, A., and Ichijo, H. (2002). ASK1 is essential for endoplasmic reticulum stress-induced neuronal cell death triggered by expanded polyglutamine repeats. *Genes & development* 16**,** 1345-1355.

Nishitoh, H., Saitoh, M., Mochida, Y., Takeda, K., Nakano, H., Rothe, M., Miyazono, K., and Ichijo, H. (1998). ASK1 is essential for JNK/SAPK activation by TRAF2. *Molecular cell* 2**,** 389-395.

Nishizuka, Y. (1984). The role of protein kinase C in cell surface signal transduction and tumour promotion. *Nature* 308**,** 693.

Novoa, I., Zeng, H., Harding, H.P., and Ron, D. (2001). Feedback inhibition of the unfolded protein response by GADD34-mediated dephosphorylation of eIF2α. *The Journal of cell biology* 153**,** 1011-1022.

Novoa, I., Zhang, Y., Zeng, H., Jungreis, R., Harding, H.P., and Ron, D. (2003). Stress‐induced gene expression requires programmed recovery from translational repression. *The EMBO journal* 22**,** 1180-1187.

Nusser, N., Gosmanova, E., Zheng, Y., and Tigyi, G. (2002). Nerve growth factor signals through TrkA, phosphatidylinositol 3-kinase, and Rac1 to inactivate RhoA during the initiation of neuronal differentiation of PC12 cells. *Journal of biological chemistry* 277**,** 35840-35846.

Obata, K., Yamanaka, H., Fukuoka, T., Yi, D., Tokunaga, A., Hashimoto, N., Yoshikawa, H., and Noguchi, K. (2003). Contribution of injured and uninjured dorsal root ganglion neurons to pain behavior and the changes in gene expression following chronic constriction injury of the sciatic nerve in rats. *Pain* 101**,** 65-77.

Ohoka, N., Yoshii, S., Hattori, T., Onozaki, K., and Hayashi, H. (2005). TRB3, a novel ER stress‐inducible gene, is induced via ATF4–CHOP pathway and is involved in cell death. *The EMBO journal* 24**,** 1243-1255.

Ortega, F., Pérez-Sen, R., Morente, V., Delicado, E.G., and Miras-Portugal, M.T. (2010). P2X7, NMDA and BDNF receptors converge on GSK3 phosphorylation and cooperate to promote survival in cerebellar granule neurons. *Cellular and molecular life sciences* 67**,** 1723-1733.

Oyadomari, S., and Mori, M. (2004). Roles of CHOP/GADD153 in endoplasmic reticulum stress. *Cell death and differentiation* 11**,** 381.

Ozes, O.N., Mayo, L.D., Gustin, J.A., Pfeffer, S.R., Pfeffer, L.M., and Donner, D.B. (1999). NF-κB activation by tumour necrosis factor requires the Akt serine–threonine kinase. *Nature* 401**,** 82.

Pinton, P., Giorgi, C., Siviero, R., Zecchini, E., and Rizzuto, R. (2008). Calcium and apoptosis: ER-mitochondria Ca 2+ transfer in the control of apoptosis. *Oncogene* 27**,** 6407.

Plongthongkum, N., Kullawong, N., Panyim, S., and Tirasophon, W. (2007). Ire1 regulated XBP1 mRNA splicing is essential for the unfolded protein response (UPR) in Drosophila melanogaster. *Biochemical and biophysical research communications* 354**,** 789-794.

Pomerantz, J., Schreiber-Agus, N., Liégeois, N.J., Silverman, A., Alland, L., Chin, L., Potes, J., Chen, K., Orlow, I., and Lee, H.-W. (1998). The Ink4a tumor suppressor gene product, p19Arf, interacts with MDM2 and neutralizes MDM2's inhibition of p53. *Cell* 92**,** 713-723.

Proud, C.G. (Year). "eIF2 and the control of cell physiology", in: *Seminars in cell & developmental biology*: Elsevier), 3-12.

Qi, Y., Gregory, M.A., Li, Z., Brousal, J.P., West, K., and Hann, S.R. (2004). p19 ARF directly and differentially controls the functions of c-Myc independently of p53. *Nature* 431**,** 712.

Raad, H., Paclet, M.-H., Boussetta, T., Kroviarski, Y., Morel, F., Quinn, M.T., Gougerot-Pocidalo, M.-A., Dang, P.M.-C., and El-Benna, J. (2009). Regulation of the phagocyte NADPH oxidase activity: phosphorylation of gp91phox/NOX2 by protein kinase C enhances its diaphorase activity and binding to Rac2, p67phox, and p47phox. *The FASEB Journal* 23**,** 1011-1022.

Radeke, M.J., Misko, T.P., Hsu, C., Herzenberg, L.A., and Shooter, E.M. (1987). Gene transfer and molecular cloning of the rat nerve growth factor receptor. *Nature* 325**,** 593-597.

Raingeaud, J., Whitmarsh, A.J., Barrett, T., Derijard, B., and Davis, R.J. (1996). MKK3-and MKK6-regulated gene expression is mediated by the p38 mitogen-activated protein kinase signal transduction pathway. *Molecular and cellular biology* 16**,** 1247-1255.

Rao, R.V., Ellerby, H., and Bredesen, D.E. (2004). Coupling endoplasmic reticulum stress to the cell death program. *Cell death and differentiation* 11**,** 372.

Rawlings, J.S., Rosler, K.M., and Harrison, D.A. (2004). The JAK/STAT signaling pathway. *Journal of cell science* 117**,** 1281-1283.

Roberts, P.J., and Der, C.J. (2007). Targeting the Raf-MEK-ERK mitogen-activated protein kinase cascade for the treatment of cancer. *Oncogene* 26**,** 3291.

Rodriguez-Tebar, A., Dechant, G., and Barde, Y.-A. (1990). Binding of brain-derived neurotrophic factor to the nerve growth factor receptor. *Neuron* 4**,** 487-492.

Rössig, L., Jadidi, A.S., Urbich, C., Badorff, C., Zeiher, A.M., and Dimmeler, S. (2001). Akt-dependent phosphorylation of p21Cip1 regulates PCNA binding and proliferation of endothelial cells. *Molecular and cellular biology* 21**,** 5644-5657.

Rouse, J., Cohen, P., Trigon, S., Morange, M., Alonso-Llamazares, A., Zamanillo, D., Hunt, T., and Nebreda, A.R. (1994). A novel kinase cascade triggered by stress and heat shock that stimulates MAPKAP kinase-2 and phosphorylation of the small heat shock proteins. *Cell* 78**,** 1027-1037.

Schlessinger, J., and Ullrich, A. (1992). Growth factor signaling by receptor tyrosine kinases. *Neuron* 9**,** 383-391.

Scott, F.L., Denault, J.B., Riedl, S.J., Shin, H., Renatus, M., and Salvesen, G.S. (2005). XIAP inhibits caspase‐3 and‐7 using two binding sites: evolutionarily conserved mechanism of IAPs. *The EMBO journal* 24**,** 645-655.

Sharma, M., Chuang, W.W., and Sun, Z. (2002). Phosphatidylinositol 3-kinase/Akt stimulates androgen pathway through GSK3β inhibition and nuclear β-catenin accumulation. *Journal of Biological Chemistry* 277**,** 30935-30941.

Shen, J., Chen, X., Hendershot, L., and Prywes, R. (2002). ER stress regulation of ATF6 localization by dissociation of BiP/GRP78 binding and unmasking of Golgi localization signals. *Developmental cell* 3**,** 99-111.

Shen, K., and Meyer, T. (1999). Dynamic control of CaMKII translocation and localization in hippocampal neurons by NMDA receptor stimulation. *Science* 284**,** 162-167.

Sherr, C.J., and Roberts, J.M. (1999). CDK inhibitors: positive and negative regulators of G1-phase progression. *Genes & development* 13**,** 1501-1512.

Shohami, E., Gallily, R., Mechoulam, R., Bass, R., and Ben-Hur, T. (1997). Cytokine production in the brain following closed head injury: dexanabinol (HU-211) is a novel TNF-α inhibitor and an effective neuroprotectant. *Journal of neuroimmunology* 72**,** 169-177.

Slee, E.A., Harte, M.T., Kluck, R.M., Wolf, B.B., Casiano, C.A., Newmeyer, D.D., Wang, H.-G., Reed, J.C., Nicholson, D.W., and Alnemri, E.S. (1999). Ordering the cytochrome c–initiated caspase cascade: hierarchical activation of caspases-2,-3,-6,-7,-8, and-10 in a caspase-9–dependent manner. *The Journal of cell biology* 144**,** 281-292.

Smith, M.L., Chen, I.-T., Zhan, Q., Bae, I., Chen, C.-Y., Gilmer, T.M., Kastan, M.B., O'connor, P.M., and Fornace, A. (1994). Interaction of the p53-regulated protein Gadd45 with proliferating cell nuclear antigen. *Science* 266**,** 1376-1380.

Soucie, E.L., Annis, M.G., Sedivy, J., Filmus, J., Leber, B., Andrews, D.W., and Penn, L.Z. (2001). Myc potentiates apoptosis by stimulating Bax activity at the mitochondria. *Molecular and cellular biology* 21**,** 4725-4736.

Srinivasula, S.M., Hegde, R., Saleh, A., Datta, P., Shiozaki, E., Chai, J., Lee, R.-A., Robbins, P.D., Fernandes-Alnemri, T., and Shi, Y. (2001). A conserved XIAP-interaction motif in caspase-9 and Smac/DIABLO regulates caspase activity and apoptosis. *Nature* 410**,** 112.

Srivastava, R.K., Li, C., Ahmad, A., Abrams, O., Gorbatyuk, M.S., Harrod, K.S., Wek, R.C., Afaq, F., and Athar, M. (2016). ATF4 regulates arsenic trioxide-mediated NADPH oxidase, ER-mitochondrial crosstalk and apoptosis. *Archives of biochemistry and biophysics* 609**,** 39-50.

Staller, P., Peukert, K., Kiermaier, A., Seoane, J., Lukas, J., Karsunky, H., Möröy, T., Bartek, J., Massagué, J., and Hänel, F. (2001). Repression of p15 INK4b expression by Myc through association with Miz-1. *Nature cell biology* 3**,** 392.

Stambolic, V., Macpherson, D., Sas, D., Lin, Y., Snow, B., Jang, Y., Benchimol, S., and Mak, T. (2001). Regulation of PTEN transcription by p53. *Molecular cell* 8**,** 317-325.

Stehlik, C., De Martin, R., Kumabashiri, I., Schmid, J.A., Binder, B.R., and Lipp, J. (1998). Nuclear factor (NF)-κB–regulated X-chromosome–linked iap gene expression protects endothelial cells from tumor necrosis factor α–induced apoptosis. *Journal of Experimental Medicine* 188**,** 211-216.

Stokoe, D., Campbell, D., Nakielny, S., Hidaka, H., Leevers, S., Marshall, C., and Cohen, P. (1992). MAPKAP kinase‐2; a novel protein kinase activated by mitogen‐activated protein kinase. *The EMBO journal* 11**,** 3985-3994.

Suzuki, Y., Imai, Y., Nakayama, H., Takahashi, K., Takio, K., and Takahashi, R. (2001). A serine protease, HtrA2, is released from the mitochondria and interacts with XIAP, inducing cell death. *Molecular cell* 8**,** 613-621.

Tajeddine, N. (2016). How do reactive oxygen species and calcium trigger mitochondrial membrane permeabilisation? *Biochimica et Biophysica Acta (BBA)-General Subjects* 1860**,** 1079-1088.

Tamatani, M., Che, Y.H., Matsuzaki, H., Ogawa, S., Okado, H., Miyake, S.-I., Mizuno, T., and Tohyama, M. (1999). Tumor necrosis factor induces Bcl-2 and Bcl-x expression through NFκB activation in primary hippocampal neurons. *Journal of Biological Chemistry* 274**,** 8531-8538.

Tanaka, C., and Nishizuka, Y. (1994). The protein kinase C family for neuronal signaling. *Annual review of neuroscience* 17**,** 551-567.

Tibbles, L.A., Ing, Y., Kiefer, F., Chan, J., Iscove, N., Woodgett, J., and Lassam, N. (1996). MLK‐3 activates the SAPK/JNK and p38/RK pathways via SEK1 and MKK3/6. *The EMBO journal* 15**,** 7026-7035.

Walter, P., and Ron, D. (2011). The unfolded protein response: from stress pathway to homeostatic regulation. *Science* 334**,** 1081-1086.

Wang, P., Qiu, W., Dudgeon, C., Liu, H., Huang, C., Zambetti, G., Yu, J., and Zhang, L. (2009). PUMA is directly activated by NF-κB and contributes to TNF-α-induced apoptosis. *Cell death and differentiation* 16**,** 1192.

Wang, X., and Ron, D. (1996). Stress-induced phosphorylation and activation of the transcription factor CHOP (GADD153) by p38 MAP kinase. *Science* 272**,** 1347-1349.

Wee, K.B., Surana, U., and Aguda, B.D. (2009). Oscillations of the p53-Akt network: implications on cell survival and death. *PloS one* 4**,** e4407.

Weinberg, R.A. (1995). The retinoblastoma protein and cell cycle control. *Cell* 81**,** 323-330.

Wek, R., Jiang, H.-Y., and Anthony, T. (2006). "Coping with stress: eIF2 kinases and translational control". Portland Press Limited).

Whitfield, J., Neame, S.J., Paquet, L., Bernard, O., and Ham, J. (2001). Dominant-negative c-Jun promotes neuronal survival by reducing BIM expression and inhibiting mitochondrial cytochrome c release. *Neuron* 29**,** 629-643.

Wong, W.W., Gentle, I., Nachbur, U., Anderton, H., Vaux, D., and Silke, J. (2010). RIPK1 is not essential for TNFR1-induced activation of NF-κB. *Cell death and differentiation* 17**,** 482.

Xing, J., Ginty, D.D., and Greenberg, M.E. (1996). Coupling of the RAS-MAPK pathway to gene activation by RSK2, a growth factor-regulated CREB kinase. *Science* 273**,** 959-963.

Xiong, Y., Hannon, G.J., Zhang, H., Casso, D., Kobayashi, R., and Beach, D. (1993). p21 is a universal inhibitor of cyclin kinases. *nature* 366**,** 701.

Xu, Z., Maroney, A.C., Dobrzanski, P., Kukekov, N.V., and Greene, L.A. (2001). The MLK family mediates c-Jun N-terminal kinase activation in neuronal apoptosis. *Molecular and cellular biology* 21**,** 4713-4724.

Yamaguchi, H., Bhalla, K., and Wang, H.-G. (2003). Bax plays a pivotal role in thapsigargin-induced apoptosis of human colon cancer HCT116 cells by controlling Smac/Diablo and Omi/HtrA2 release from mitochondria. *Cancer research* 63**,** 1483-1489.

Yamaguchi, K., Nagai, S.I., Ninomiya‐Tsuji, J., Nishita, M., Tamai, K., Irie, K., Ueno, N., Nishida, E., Shibuya, H., and Matsumoto, K. (1999). XIAP, a cellular member of the inhibitor of apoptosis protein family, links the receptors to TAB1–TAK1 in the BMP signaling pathway. *The EMBO journal* 18**,** 179-187.

Yamashita, M., Fatyol, K., Jin, C., Wang, X., Liu, Z., and Zhang, Y.E. (2008). TRAF6 mediates Smad-independent activation of JNK and p38 by TGF-β. *Molecular cell* 31**,** 918-924.

Yamauchi, K., Osuka, K., Takayasu, M., Usuda, N., Nakazawa, A., Nakahara, N., Yoshida, M., Aoshima, C., Hara, M., and Yoshida, J. (2006). Activation of JAK/STAT signalling in neurons following spinal cord injury in mice. *Journal of neurochemistry* 96**,** 1060-1070.

Yang, E., Zha, J., Jockel, J., Boise, L.H., Thompson, C.B., and Korsmeyer, S.J. (1995). Bad, a heterodimeric partner for Bcl-XL and Bcl-2, displaces Bax and promotes cell death. *Cell* 80**,** 285-291.

Yang, L., Sun, M., Sun, X.-M., Cheng, G.Z., Nicosia, S.V., and Cheng, J.Q. (2007). Akt attenuation of the serine protease activity of HtrA2/Omi through phosphorylation of serine 212. *Journal of Biological Chemistry* 282**,** 10981-10987.

Yang, X., Khosravi-Far, R., Chang, H.Y., and Baltimore, D. (1997). Daxx, a novel Fas-binding protein that activates JNK and apoptosis. *Cell* 89**,** 1067-1076.

Zareen, N., Biswas, S.C., and Greene, L.A. (2013). A feed-forward loop involving Trib3, Akt and FoxO mediates death of NGF-deprived neurons. *Cell Death Differ* 20**,** 1719-1730.

Zhang, Q.G., Wang, X.T., Han, D., Yin, X.H., Zhang, G.Y., and Xu, T.L. (2006). Akt inhibits MLK3/JNK3 signaling by inactivating Rac1: a protective mechanism against ischemic brain injury. *Journal of neurochemistry* 98**,** 1886-1898.

Zhao, L., Samuels, T., Winckler, S., Korgaonkar, C., Tompkins, V., Horne, M.C., and Quelle, D.E. (2003). Cyclin G1 Has Growth Inhibitory Activity Linked to the ARF-Mdm2-p53 and pRb Tumor Suppressor Pathways1 1 DEQ from the American Cancer Society (RSG-98-254-04-MGO) and NIH (RO1 CA90367), and by a grant from the NIH to MCH (RO1 GM56900). *Molecular Cancer Research* 1**,** 195-206.

Zhu, Y., Mao, X.O., Sun, Y., Xia, Z., and Greenberg, D.A. (2002). p38 Mitogen-activated protein kinase mediates hypoxic regulation of Mdm2 and p53 in neurons. *Journal of Biological Chemistry* 277**,** 22909-22914.

Zindy, F., Eischen, C.M., Randle, D.H., Kamijo, T., Cleveland, J.L., Sherr, C.J., and Roussel, M.F. (1998). Myc signaling via the ARF tumor suppressor regulates p53-dependent apoptosis and immortalization. *Genes & development* 12**,** 2424-2433.
